# Supplementary material for: Study protocol for a randomised controlled trial assessing the impact of pulmonary rehabilitation on maximal exercise capacity for adults living with post-TB lung disease: Global RECHARGE Uganda
Source: BMJ Open. 2021 Aug 10;11(8):e047641. doi: 10.1136/bmjopen-2020-047641 (PMC8356159; doi:10.1136/bmjopen-2020-047641)
Supplement: Supplementary data [file bmjopen-2020-047641supp001.pdf]

Supplementary Table 1: A list of warm up and cool down activities during PR session

1. Marching on the spot, slowly bringing the feet off the floor for up to 1 minute
2. Heel digs: alternate heel digs in front of the body with toes pointing to the ceiling, add in a biceps curl (repeat 10-12 times).
3. Toe taps: Tap the toes to the floor in front of alternating legs at a comfortable distance. Heels stay off the ground (repeat 10-12 times).
4. High knee marching – with opposite hand to opposite knee.
5. Side bends: With arms relaxed by your side, leaning over to the right for 8 to 10 seconds and back to centre, then lean to the left for 8 to 10 seconds and back to the centre (repeat 8 times).
6. Arms stretched up, forwards and down.
7. Alternate punching of arms forward.
8. Seated or standing side taps to the floor with the foot – alternate legs.
9. Seated or standing in upright posture, feet placed shoulder breadth apart – shoulder roll in both directions (clockwise and anti-clockwise).
10. Seated or standing in upright posture, feet placed shoulder breath apart, elbows bent with hands onto shoulder – elbows make circles in clockwise and anti-clockwise.
11. Hamstring stretch: With right leg straight, place it in front of the body, heel pushed into the floor with toes pointing toward the ceiling. Slightly bend the left knee, place hands on the straight right leg and gently lean forward. Hold the stretch for 10-15 seconds then return to upright position. Repeat on left leg.
12. Quadriceps stretch: While holding a chair or onto a wall, stand on your left leg and grab your right foot using your right hand, pulling it gently towards the ceiling. Hold the position for 10-15 seconds and return to upright position and repeat on the right leg.

PR – Pulmonary Rehabilitation
