# Supplementary material for: Study protocol for a randomised controlled trial assessing the impact of pulmonary rehabilitation on maximal exercise capacity for adults living with post-TB lung disease: Global RECHARGE Uganda
Source: BMJ Open. 2021 Aug 10;11(8):e047641. doi: 10.1136/bmjopen-2020-047641 (PMC8356159; doi:10.1136/bmjopen-2020-047641)
Supplement: Supplementary data [file bmjopen-2020-047641supp002.pdf]

Supplementary Table 2: Physical activity data collection and accelerometry processing criteria

Criteria	Details
**Accelerometer Model**	ActiGraph wGT3X-BT (version 6.13.4; firmware 1.9.2)
**Serial number range**	Twenty unique devices will be used; ranging from MOS2E09190617 to MOS2E25190750 and averaging six deployments per device (same serial used for baseline and follow-up wear periods to remove any inter-device variability)
**Piezosensor orientation**	Triaxial
**Mode setup**	Mode 29 (x, y, z, steps, lux)
**Original sample rate**	100 Hz (.gt3x file format)
**Deployment method**	Baseline:<br>Fitted by research team on Day 0 (Baseline PR Assessment)<br>Fitted by participant on Day 1<br>Follow-up:<br>Fitted by research team Day 0 (11th/12th session)<br>Fitted by participant on Day 1
**Location worn**	Anterior hip adjacent to the mid-line of the thigh
**Requested days of wear**	7 days of free-living (10,080 epochs)
**Initialization**	Not deployed in delay mode in order to standardised capture of Day 0 (00:00) with stop time based on date of first PR class (baseline) and date of follow-up assessment
**Wear instructions**	Wear continuously except for sleep and water-based activities
**Non-wear appropriation**	≥60 min of consecutive 0s with allowance for 2 minutes of interruptions
**Valid day criteria**	≥8 hours of valid waking wear time
**Valid file**	≥4 valid days for each of the two time points
**Missing data**	Data modelling or imputation will not be performed
**Epoch length**	60 seconds
**Intensity classification (absolute)**	Uniaxial (x-axis) intensity cut-points as follows: Stationary <100 cpm; Light 100-2019 cpm; Moderate 2020-5998cpm; Vigorous ≥5999cpm (Moderate-to-vigorous ≥2020 cpm)
**Intensity classification (relative)**	Uniaxial (x-axis) cut-points based on Endurance Shuttle Walk Test performance

PR – Pulmonary Rehabilitation
