# Supplementary material for: Study protocol for a randomised controlled trial assessing the impact of pulmonary rehabilitation on maximal exercise capacity for adults living with post-TB lung disease: Global RECHARGE Uganda
Source: BMJ Open. 2021 Aug 10;11(8):e047641. doi: 10.1136/bmjopen-2020-047641 (PMC8356159; doi:10.1136/bmjopen-2020-047641)
Supplement: Author's manuscript [file bmjopen-2020-047641.draft_revisions.pdf]

BMJ Open is committed to open peer review. As part of this commitment we make the peer review history of every article we publish publicly available.

When an article is published we post the peer reviewers' comments and the authors' responses online. We also post the versions of the paper that were used during peer review. These are the versions that the peer review comments apply to.

The versions of the paper that follow are the versions that were submitted during the peer review process. They are not the versions of record or the final published versions. They should not be cited or distributed as the published version of this manuscript.

BMJ Open is an open access journal and the full, final, typeset and author-corrected version of record of the manuscript is available on our site with no access controls, subscription charges or pay-per-view fees (http://bmjopen.bmj.com).

If you have any questions on BMJ Open's open peer review process please email info.bmjopen@bmj.com

BMJ Open

A study protocol for a randomised controlled trial assessing the impact of Pulmonary Rehabilitation on maximal exercise capacity for adults living with Post-TB lung disease: Global RECHARGE Uganda

Journal:	*BMJ Open*
Manuscript ID	bmjopen-2020-047641
Article Type:	Protocol
Date Submitted by the Author:	07-Dec-2020
Complete List of Authors:	Katagira, Winceslaus; Makerere University, Lung Institute<br>Orme, Mark; University of Leicester, Respiratory Sciences<br>Jones, Amy; University of Leicester, Respiratory Sciences<br>Kasiita, Richard; Mulago Hospital, Kampala, Uganda, Department of Physiotherapy,<br>Rupert, Jones; University of Plymouth (UK), Faculty of Health<br>Barton, Andy; University of Plymouth (UK), Faculty of Health<br>Miah, Ruhme ; University of Leicester, Department of Respiratory Sciences<br>Manise, Adrian ; University of Leicester, Department of Respiratory Sciences<br>Matheson, Jesse; University of Sheffield, Department of Economics<br>Free, Robert; Centre for Exercise and Rehabilitation Science (CERS), NIHR Leicester Biomedical Research Centre – Respiratory, University Hospitals of Leicester NHS Trust<br>Steiner, michael ; Institute for Lung Health, NIHR Leicester Biomedical Research Centre – Respiratory, University of Leicester, Glenfield Hospital,<br>Kirenga, Bruce; Makerere University, Lung Institute<br>Singh, Sally; University Hospitals of Leicester NHS Trust, Cardiac/Pulmonary Rehabilitation
Keywords:	Rehabilitation medicine < INTERNAL MEDICINE, Health economics < HEALTH SERVICES ADMINISTRATION & MANAGEMENT, Tuberculosis < INFECTIOUS DISEASES, Adult thoracic medicine < THORACIC MEDICINE

SCHOLARONE™
Manuscripts

For peer review only - http://bmjopen.bmj.com/site/about/guidelines.xhtml

I, the Submitting Author has the right to grant and does grant on behalf of all authors of the Work (as defined in the below author licence), an exclusive licence and/or a non-exclusive licence for contributions from authors who are: i) UK Crown employees; ii) where BMJ has agreed a CC-BY licence shall apply, and/or iii) in accordance with the terms applicable for US Federal Government officers or employees acting as part of their official duties; on a worldwide, perpetual, irrevocable, royalty-free basis to BMJ Publishing Group Ltd ("BMJ") its licensees and where the relevant Journal is co-owned by BMJ to the co-owners of the Journal, to publish the Work in this journal and any other BMJ products and to exploit all rights, as set out in our licence.

The Submitting Author accepts and understands that any supply made under these terms is made by BMJ to the Submitting Author unless you are acting as an employee on behalf of your employer or a postgraduate student of an affiliated institution which is paying any applicable article publishing charge ("APC") for Open Access articles. Where the Submitting Author wishes to make the Work available on an Open Access basis (and intends to pay the relevant APC), the terms of reuse of such Open Access shall be governed by a Creative Commons licence – details of these licences and which Creative Commons licence will apply to this Work are set out in our licence referred to above.

Other than as permitted in any relevant BMJ Author's Self Archiving Policies, I confirm this Work has not been accepted for publication elsewhere, is not being considered for publication elsewhere and does not duplicate material already published. I confirm all authors consent to publication of this Work and authorise the granting of this licence.

For peer review only

Title: A study protocol for a randomised controlled trial assessing the impact of Pulmonary Rehabilitation on maximal exercise capacity for adults living with Post-TB lung disease: Global RECHARGE Uganda

Authors

Winceslaus Katagira[1], Mark W. Orme[2,3], Amy V. Jones[2,3], Richard Kasiita[4], Rupert Jones[5], Andy Barton[5], Ruhme Miah[2,3], Adrian Manise[6], Jesse Matheson[7], Robert C. Free[3], Michael C. Steiner[2,3], Bruce Kirenga[1] and Sally J. Singh[2,3]

Affiliations

1. Makerere University Lung Institute, Kampala, Uganda
2. Department of Respiratory Sciences, University of Leicester (UK)
3. Centre for Exercise and Rehabilitation Science (CERS), NIHR Leicester Biomedical Research Centre – Respiratory, University Hospitals of Leicester NHS Trust - Leicester (UK)
4. Department of Physiotherapy, Mulago Hospital, Kampala, Uganda
5. Faculty of Health, University of Plymouth (UK)
6. NIHR Leicester Biomedical Research Centre, University Hospitals of Leicester NHS Trust - Leicester (UK)
7. Department of Economics, University of Sheffield, Sheffield, United Kingdom

Protocol version:

Version 1.0 July 2019

Funding source:

This research was funded by the National Institute for Health Research (NIHR) (17/63/20) using UK aid from the UK Government to support global health research. The views expressed in this publication are those of the author(s) and not necessarily those of the NIHR or the UK Department of Health and Social Care.

Roles and responsibilities of authors

a) Substantially contributed to the conception and/ or design of the work
b) Substantially contributed to the acquisition, analysis, or interpretation of data for the work
c) Substantially contributed to the drafting of the work and/ or revising it critically for important intellectual content

Name and contact information for corresponding author

Katagira Winceslaus, wincegira@gmail.com

Word count: 3,998

For peer review only

Abstract

Introduction

The burden of post-TB lung disease (PTBLD) is steadily increasing in sub-Saharan Africa, causing disability among TB survivors. Without effective medicines, the mainstay of PTBLD treatment evolves around disease prevention and supportive treatment. Pulmonary rehabilitation (PR), a low-cost, non-pharmacological intervention has shown effectiveness in a group of PTBLD individuals but has not been tested in a clinical trial. This study aims to assess the impact of a 6-week PR programme on maximal exercise capacity and other outcomes among adults in Uganda living with PTBLD.

Methods and analysis

This is a randomized waiting-list controlled trial with blinded outcome measures, comparing PR versus usual care for patients with PTBLD. A total of 114 participants will be randomized (1:1) to receive either usual care (on the waiting list) or PR, with follow-up assessments at 6- and 12-weeks post-intervention. The primary outcome is change in walking distance measured by the Incremental Shuttle Walk Test from baseline to the end of 6-weeks of PR. All secondary outcomes will be compared between the PR and usual care arms from baseline to 6- and 12-week follow-ups. Secondary outcomes include self-reported respiratory symptoms, physical activity, psychological well-being, health related quality of life and cost benefit analysis. All randomized participants will be included in the intention to treat analysis population. The primary efficacy analysis will be based on both per protocol and modified intention-to-treat populations.

Ethics and dissemination

The trial has received ethical clearance from the Mulago Hospital Research and Ethics Committee (MHREC 1478), Kampala, Uganda as well as the Uganda National Council for Science and Technology (SS 5105). Ethical approval has been obtained from the University of Leicester, United Kingdom research ethics committee (Ref No. 22349). Study findings will be published in appropriate peer-reviewed journals and disseminated at appropriate local, regional and international scientific meetings and conferences.

Strengths and limitations of this study

The study aims to determine the effectiveness of PR for individuals with PTBLD in a clinical trial setting. To our knowledge, this is the first pragmatic, fully powered effectiveness trial for

For peer review only - http://bmjopen.bmj.com/site/about/guidelines.xhtml

PR in PTBLD in Africa. This is a progression of previous work that established feasibility and acceptability of PR design for people living with PTBLD in Uganda.

Due to funding limitations, we are unable to carry out a multi-site study. This may limit generalizability of the study findings.

Trial registration: ISRCTN18256843

Key words

Pulmonary Rehabilitation, Post-TB lung disease, Chronic Respiratory disease, non-drug treatment, Exercise, Education

Introduction

Background and rationale

In 2018, 24% of the global Tuberculosis (TB) incident cases occurred in the African region [1]. Furthermore, 24 of the 30 high TB/Human Immunodeficiency Virus (HIV) burden countries, including Uganda, are in the African region; accounting for 71% of the global burden of HIV associated TB [1]. Despite great strides made over the recent years to achieve the 90% treatment success rate, as part of the "End TB strategy" target [1], a significant number of TB survivors continue to have poor health related quality of life [2]. This may be attributed to the pulmonary function impairment following TB treatment which has been reported in approximately 50% of pulmonary TB survivors [3]. The reduction in ventilation and perfusion attributed to the permanent lung parenchymal damage [4] clinically manifests as long-term respiratory symptoms and eventually chronic respiratory disease (CRD), including chronic obstructive pulmonary disease (COPD), bronchiectasis and aspergillosis [5, 6].

Adults with post-TB respiratory symptoms develop skeletal muscle dysfunction, related to physical inactivity and systemic inflammation, which is often compounded by impaired nutrition and poverty [7]. Such patients enter a vicious cycle with falling body weight, progressive morbidity and increased mortality [7]. Individuals affected by CRDs tend to avoid exercise and become increasingly deconditioned and demotivated, leading to a cycle of decline. There are no effective medicines for post-TB lung disease (PTBLD) and the mainstay of

For peer review only - http://bmjopen.bmj.com/site/about/guidelines.xhtml

treatment evolves around disease prevention and supportive treatment. The disease, previously neglected by health services and researchers, is now the focus of increasing interest [8, 9].

In low- and middle-income countries (LMIC) where health care focuses on treatment and prevention of infectious diseases, as opposed to managing chronic diseases, the care for adults living with CRD presents a major challenge. Consequently, patients that require long-term and systemic approaches often receive sub-optimal medical care, inevitably leading to preventable deaths in resource poor settings.

Pulmonary Rehabilitation (PR) is a low cost, high impact intervention that reverses the disability associated with CRDs, and is supported by the highest level of research evidence in high income countries [10, 11]. A PR program brings together health professionals from many disciplines offering supervised exercise training and disease education, supporting people to manage their own disease. However, in LMIC where the burden of CRDs is increasing fastest, PR is scarce and health care services are poorly adapted to deal with such diseases. Although PR is a grade "A" evidence treatment for adults with COPD [12] and has been utilized in other chronic lung diseases [13], it's efficacy in Post TB lung disease is not known. In a development study to examine the impact of PR for people with PTBLD in Uganda, it was feasible to run a PR programme and participants reported clinically important improvements in quality of life, exercise capacity, and respiratory outcomes [14]. To date, there has been little attention to the role of PR in PTBLD globally, particularly in Africa where a significant number of PTB survivors reside.

Study Objectives

The primary objective of this trial is to assess the impact of a 6-week PR programme on maximal exercise capacity using the incremental shuttle walking test (ISWT) among adults living with PTBLD post-intervention.

The secondary objectives include assessing the impact of PR on quality of life and other outcomes for patients with Post-TB lung disease, and to conduct a cost-benefit analysis of PR.

Methods

Study design

This is a prospective, randomised waiting-list controlled trial with blinded outcome measures, comparing PR versus usual care for patients with post-TB lung disease. During this

For peer review only - http://bmjopen.bmj.com/site/about/guidelines.xhtml

effectiveness trial, a total of 114 participants will be randomized (1:1) to receive either usual care (waiting-list) or PR (Figure 1).

Study setting

The study is conducted at the PR centre located at the Makerere University Lung Institute (MLI) Clinic, Kampala, Uganda. The MLI clinic is an academic outpatient clinic within the Mulago National Referral hospital, a teaching and clinical research hospital for Makerere University.

Study population

Recruitment

Adults with PTBLD will be referred from health facilities and clinics (TB treatment centres and HIV/TB caring centres) around Kampala to the PR centre. Existing registers have around 300 adults living with PTBLD and additional patients will be screened directly from the outpatient departments.

Participant invitation

The process of identifying and inviting eligible patients was refined in the development study. Eligible individuals identified as having an established PTBLD diagnosis will be received at the PR centre at the MLI. Literate participants will be asked to read the patient information sheet (PIS) about the study, written in English or translated in the local language. Illiterate participants will have the contents read to them in full by a study staff, in the presence of a witness who will be present during the whole process. Participants will have the opportunity to discuss the PIS with the study medical personnel. Once the study staff are satisfied that the participant has understood the PIS, and is interested in taking part in the study, they will be taken through the informed consent process. Participants will give consent before undergoing screening tests and procedures, and if still eligible after the screening process, will be taken through another informed consent process for randomisation.

Eligibility criteria

Inclusion criteria

A patient with PTBLD is eligible for the trial if they meet all of the following criteria: aged ≥18 years, willing and able to provide written informed consent (signed or witnessed consent if the patient is illiterate), a documented past history of smear positive pulmonary TB with

For peer review only - http://bmjopen.bmj.com/site/about/guidelines.xhtml

treatment completed ≥6 months prior to study enrolment, a negative Xpert MTB/RIF assay for *Mycobacterium tuberculosis* at the time of study enrolment, and report a Medical Research Council (MRC) dyspnoea grade ≥2.

Exclusion criteria

A PTBLD patient is ineligible for the study if they have co-morbidities that preclude exercise (e.g. known unstable cardiovascular disease, locomotor difficulties) or if they are unwilling to participate for any reason or had any condition (social or medical) which in the opinion of the investigator would make study participation unsafe.

Randomization

Once eligible participants have consented to take part in the study, they will be randomised using a web-based randomisation system (https://www.sealedenvelope.com/). Participants will be randomized (1:1) to receive either usual care or PR. Access to the web-based system will be controlled through an authorised username and password. Randomisations will be conducted by a member of the study team independent from the data collection team and will be revealed to the data collection and intervention delivery teams after baseline measurements have been obtained.

Participant timeline

After randomisation, the PR team will explain to participants when the PR sessions will take place. For each individual participant, the hospital based PR programme will last six weeks followed by a follow-up period of six weeks of home exercises. Participants in the control arm (waiting-list) of the trial will be informed of the date for their first exercise session in approximately 12-15 weeks. Based on our development study [15], we expect to find prolonged and possibly improved effects of PR at follow-up. Our experience indicates that a follow-up period of more than three months after the start of the PR programme would be unrealistic in this environment without unacceptable attrition. Study participants will receive compensation for their time and transport.

Pulmonary Rehabilitation Team

The PR team has received adequate training on the delivery of PR and participated in the development study which informed this trial [14]. Furthermore, the individuals are registered health professionals (physiotherapist, physicians, and nurses) and have undertaken training regarding the study tests, procedures and measurements per protocol as well as Good Clinical Practice.

For peer review only

Assessment and follow up

Participants in both arms of the trial will be asked to attend the baseline, 6-week and 12-week post-intervention assessment visits at the PR centre at MLI. Data will be collected by the study staff (medical doctor, nurse and physiotherapist). Table 1 shows all baseline and follow up assessment data that will be collected during the trial, in accordance with a minimum recommended dataset for PR trials in LMIC[16].

Study procedures

During the screening visit, prospective participants will undergo clinical examination, MRC dyspnoea grading, sputum examination using Xpert MTB/RIF assay and a frontal chest radiograph. In addition, demographic, socio-economic, medical and clinical history (including respiratory symptoms and exposure history to cigarettes and biomass) will be collected using a standardised questionnaire.

At the randomisation visit, spirometry will be performed using American Thoracic Society and European Respiratory Society guidelines [17].

Sample size

The study will be powered to detect a 35m difference in the ISWT measured at baseline and after completion of PR[18]. Assuming that ISWT follows an approximately normal distribution, a power calculation based on a paired t-test was performed. Assuming a statistical significance level of 5% and a statistical power of 80%, in order to detect a 35m difference in ISWT measured at baseline and after completion of PR, a total of 114 participants are required (PR: 57 participants or waiting list: 57 participants). Our recent feasibility study [15] was used to obtain an estimate of the pooled standard deviation for the power calculation. Conservatively assuming up to 30% loss to follow-up at 6-weeks, this will require up to 114 participants to be recruited and randomised (1:1) to each arm. Using the 70% ineligibility rate during screening from the feasibility study, we will need to screen approximately 543 PTBLD patients.

Blinding (masking)

Due to the nature of PR, it will not be possible to blind participants to their group allocation but participants will be asked not to reveal their group during the follow-up assessments. The participant and treating clinician will be aware of treatment allocation, however, the outcome measures will be performed by staff blinded to treatment allocation and the ISWT (primary

For peer review only - http://bmjopen.bmj.com/site/about/guidelines.xhtml

outcome) will be prioritised to reduce the risk of un-blinding. Any episodes of un-blinding will be documented and reported.

Treatment arms

Usual care (control arm)

The participants in the waiting-list (control) arm will receive usual care and will be offered PR after completing 12-weeks of follow-up. There are currently no guidelines for the clinical management of PTBLD both locally and internationally. Usual care will be optimised where possible and will include the following: frontal chest radiograph, spirometry to screen for airway diseases, inhalational therapies for airway disease amenable to treatment (where appropriate), antibiotic and systemic glucocorticoid therapy for infective exacerbations (where appropriate), and verbal advice to reduce exposures to risk factors such as tobacco smoking and biomass smoke.

Pulmonary Rehabilitation (trial intervention arm)

In addition to usual care described above, participants in the intervention arm will receive PR. PR will consist of a six-week programme offered to a group of up to 12 participants, with sessions occurring twice weekly for at least two hours (approximately one hour for education and one hour for exercise).

Warm-up and cool-down

Before starting exercises, participants will be taken through a group warm up session, followed by a cool down session at the end of exercises, each lasting 10-15 minutes. Warm up is aimed at readying the body for both the physical aspects of performance (increased blood flow and muscle temperature) and mental readiness for exercise whilst cool down session facilitates a smoother decline in temperature and blood flow [19] Both warm up and cool down will consist of stretching and flexibility exercises during which participants will perform both upper and lower body flexibility exercises, held for 10 to 15 seconds each (including stretching of major muscle groups such as the calves, hamstrings, quadriceps, and biceps, as well as range of motion exercises for the neck, shoulders, and trunk), 2 days/week[13]. The cool down session has the same activities of warm-up (supplementary table 2) but performed at a slower pace.

Endurance training

Each participant will go through two stations of endurance exercise; load-adjustable stationary cycling and ground-based walking stations. We shall employ an intensity of continuous exercise at each station for 10 minutes or until a Borg dyspnoea score of 4-6 (moderate to [very]

For peer review only - http://bmjopen.bmj.com/site/about/guidelines.xhtml

severe) is attained [20, 21]. Participants who may have difficulty in sustaining continuous high-intensity exercise will have interspersed periods of rest or lower intensity exercise to maximise the benefits of exercise training [13]. The walking exercise regime will be individually prescribed to participants based around their performance in the ISWT. Participants will be encouraged to walk at 85% of their maximal ISWT walking speed [22].

Strength training

Each participant will go through two stations for strengthening upper limb muscles (pull-ups and biceps curls) and two for strengthening lower limb muscles (sit-to-stand and step-up exercises). Each of the stations will include 3 sets of 8-12 repetitions. Participants will be asked to continue doing both endurance and resistance exercises at home, unsupervised.

Education sessions

A dedicated education session will be conducted at the start of each class, before the exercise regimes (Table 2; 12 sessions in total).

**Table 2: Education content of the Global RECHARGE Pulmonary Rehabilitation program**
1. Normal anatomy and physiology of the lungs
2. Pathophysiology of chronic lung disease
3. Tuberculosis and how it causes lung damage
4. Coping with chronic lung disease and coping with stress
5. Avoidance of risk factors for chronic lung disease
6. Early recognition and treatment of exacerbations
7. Strategies for managing breathlessness
8. Energy conservation during activities of daily living
9. Role and rationale for medications and devices
10. Benefit of exercise and physical activities
11. Healthy food intake
12. Secretion clearance techniques

Study Outcomes

Primary outcome

The primary outcome is change in walking distance measured by the ISWT from pre to post-intervention. A group change of at least 35m is considered clinically important [18].

For peer review only - http://bmjopen.bmj.com/site/about/guidelines.xhtml

Incremental Shuttle Walking Test

The ISWT requires the patient to walk up and down a 10-meter course, identified by two cones inset 0.5m from either end to avoid the need for abrupt changes in direction. The speed at which the patient walks is dictated by an audio signal played on an audio device. Each participant will receive standardised instructions to: "Walk at a steady pace, aiming to turn around when you hear the signal. You should continue to walk until you feel that you are unable to maintain the required speed without becoming unduly breathless" [23]. To ensure the learning effect is accounted for, a practice ISWT will be performed and the participant will receive encouragement from the physiotherapist throughout the test in an effort to increase the distance one can walk. The test is terminated when either 1) the patient indicates that they are unable to continue, 2) if the operator determines that the patient is not fit to continue, or 3) the operator assesses that the patient was unable to sustain the speed and cover the distance to the cone prior to the beep sounding [23].

Secondary outcomes

All secondary outcomes will be compared between the PR and usual care arms from baseline to 6-week and 12-week follow-ups.

Health questionnaires will be administered including COPD assessment test (CAT), Clinical COPD questionnaire (CCQ), Hospital Anxiety and Depression Scale (HADS), Patient Health Questionnaire (PHQ-9), Work Productivity and Activity Impairment (WPAI), and European Quality of Life 5-Dimensions (EQ-5D-5L). Pulmonary rehabilitation specific measurements will include the ISWT, Endurance Shuttle Walking Test (ESWT), mid upper arm circumference (MUAC) and sit-to-stand test.

Respiratory symptoms

The CCQ is a simple 10-time validated health related quality of life (HRQoL) questionnaire with good psychometric properties [24]. It consists of 10 items, each scored between 0-6, divided into three domains (symptoms, functional, mental), with higher scores representing worse HRQoL. The CCQ is responsive to PR with an estimated minimal important improvement of 0.4 [25].

The CAT is a validated, self-administered, short and simple questionnaire that measures HRQoL [26]. The CAT consists of eight items, each scored between 0-5 scored with a range of 0-40; scores of 0-10, 11-20, 21-30, 31-40 representing mild, moderate, severe or very severe

For peer review only - http://bmjopen.bmj.com/site/about/guidelines.xhtml

negative impact on HRQoL, respectively. The CAT is responsive to the effects of PR with an estimated minimal clinically important difference (MCID) of 2 points [27].

Psychological wellbeing

The HADS questionnaire is a validated, easy to use screening tool for anxiety and depression symptoms in a hospital outpatient setting [28]. The self-report rating scale is composed of 14 items with two 7-item subscales (HADS-Anxiety and HADS-Depression), both ranging from 0-21 with higher scores indicating more severe distress. The HADS is responsive to PR with estimated MCID of 2 points on each subscale [29, 30].

The PHQ-9 is a nine item, validated, short, self-administered, and positively worded questionnaire designed to measure the severity of depression over the last 2 weeks [31]. The total score ranges from 0-27, with high scores indicating high depression, specifically; no depression (0–4), mild (5–9), moderate (10–14), moderately severe (15–19), or severe depression (20–27) [31]. The PHQ-9 has an estimated MCID of 5 points [32].

Work productivity and impairment

The WPAI questionnaire is a validated instrument to measure impairments in work and activities, both paid and unpaid. The WPAI self-administered questionnaire measures time missed from work, impairment of work and regular activities due to overall health and symptoms, during the past seven days [33]. We have added two follow-up supplementary questions, following the WPAI format, to measure productivity with respect to regular household duties in low resource settings.

Health Related Quality of Life

The EQ-5D-5L questionnaire is a standardised questionnaire, developed to measure of health outcomes and defines health in terms of five dimensions: mobility, self-care, usual activities, pain or discomfort and anxiety or depression [34]. The questionnaire also contains a visual analogue scale. The EQ-5D-5L will be used to calculate patient costs per quality adjusted life year (QALY). EQ-5D-5L is responsive to change following PR, with a MCID of 0.05 (utility index) and 7.0 (visual analogue scale) [35].

Exercise capacity/ physical function

The five-repetition sit-to-stand test (FTSTS) is a commonly used functional performance measure of lower-limb strength [36]. The FTSTS measures the time taken to stand five times from a sitting position as rapidly as possible. The FTSTS is reliable, valid and responsive to PR with an estimated MCID of 1.7 seconds [37].

For peer review only - http://bmjopen.bmj.com/site/about/guidelines.xhtml

The MRC dyspnoea scale is a 5-point self-administered questionnaire based on the sensation of breathing difficulty experienced by the patient during daily life activities. The questionnaire is short, easy to use and has grades ranging from 1 (none) to 5 (almost compete incapacity), with high grades indicating high perceived respiratory disability[38]. The MRC dyspnoea scale is responsive to PR with estimated MCID of 1 points [39, 40].

The ESWT is a constant-load exercise test which measures the ability of the participant to sustain a given sub-maximal exercise capacity; the participant aims to walk at 85% of their maximal ISWT walking speed [22]. The ESWT is frequently used as an exercise tolerance outcome measure for PR. The endpoint of the test is the time the participant walks at the constant endurance speed. The test consists of pre-recorded audio signals at different frequencies giving a total of 16 walking speeds. The ESWT is responsive to PR with MCID following a 6-week PR programme between 174-279 seconds [41].

Physical activity (PA)

Participants will be asked to wear an ActiGraph wGT3X-BT activity monitor (ActiGraph, Pensacola, FL, USA), able to detect a range of PA intensities [42]. Participants will be instructed to wear the PA monitor on the right anterior hip during waking hours for one week prior to attending PR (pre-intervention) and for one week prior to their post-intervention assessment (supplementary Table 1). Written instructions to follow will be provided to the participants prior to wearing and using the PA monitors.

Cost/benefit analysis

The cost of starting and running a PR program will include single and recurrent costs (Table 3). Single payments will include the necessary costs needed to set up and run PR. Recurrent costs refer to any item with a life expectancy of ≤1 year (e.g. disposable materials) [43]. The fixed costs will be captured prior to enrolling the first participant into the PR programme and the recurrent costs will be collected at the mid-stage of recruitment. The average fixed and recurrent costs will be calculated separately.

Table 3: Table showing the variables used to calculate fixed and recurrent costs (not an exhaustive list)

Fixed costs	Recurrent costs
• Electrical equipment (laptop, printer, projector)	• Staff time to conduct PR (assessment at baseline and

For peer review only - http://bmjopen.bmj.com/site/about/guidelines.xhtml

• Equipment for PR (weights, treadmill, cycle ergometer, country-specific equipment, step-up box, chairs)<br>• Equipment for shuttle walking tests (cones, licences, stop watches, tape measure, electrical equipment to play audio)<br>• Equipment for PR assessment (height stadiometer, weight scales, sphygmomanometer, pulse oximeter, spirometer, calibration syringe, country-specific equipment)<br>• Additional safety equipment (blood glucose monitor, Oxygen cylinder holder)<br>• Miscellaneous (filing cabinets, storage units, questionnaire translations, questionnaire licences, staff uniform)<br>• Staff time (creating core PR content including educational material, exercise diaries and other necessary paperwork)	discharge, conduct PR classes, telephone calls and data entry)<br>• Disposable equipment (for blood glucose monitor, spirometer mouthpieces, nose-clips)<br>• Servicing costs (spirometer, PR equipment, specifically cycle ergometers)<br>• Miscellaneous (Oxygen cylinders, questionnaire licences, stationery (paper)<br>• Patient costs (transport and meals)

Legend: *PR Pulmonary Rehabilitation*

Patient and public involvement (PPI)

Adults with CRDs tell us how they are greatly troubled by breathlessness and express interest in attending a program that can help better manage their condition. They express interest in attending a hospital based programme that allows them to interact with fellow patients. They have additionally the programme and how it is delivered. We have also set up a PPI group at MLI that will meet regularly, and assist with disseminating results following the study.

Data analysis

All randomized participants will be included in the intention to treat analysis population. The primary efficacy analysis will be based on both per protocol and modified intention-to-treat populations. For the primary analysis, the differences in the primary outcome (walking distance on the ISWT) with the corresponding two-sided 95% confidence interval and p-value will be estimated using a stratified analysis; a p-value <0.05 will be the measure for statistical significance. Predictive analytics software (SPSS; Statistical Package for the Social Sciences)

For peer review only - http://bmjopen.bmj.com/site/about/guidelines.xhtml

will be used to analyse the data. Continuous data will be presented as mean and standard deviation or median and interquartile ranges, whilst categorical data will be presented as frequencies and percentages. All data will be assessed for normality and appropriate parametric and non-parametric tests will be used. Categorical variables between the two treatment groups will be compared using chi-square and Fisher exact test as appropriate. Continuous variables will be compared using t-test for normally distributed data and Mann-Whitney-U test for non-normally distributed data. Any baseline differences will be adjusted for. Both intention-to-treat and per-protocol analyses will be conducted after imputing any missing data. There will be no formal interim analysis of data. The final analysis will be performed when all the 114 participants have completed the last study related visit or previously withdrawn from the trial.

Data management

An Independent Data Monitoring Committee will be established at the University of Leicester, UK to review high level safety data (serious adverse events and adverse events) at least quarterly, and as needed on an ad hoc basis to ensure the continuing safety of the participants enrolled in this study.

All data collected during the trial will be entered into the Research Electronic Data Capture (REDCap) [44, 45] with access via a secure password protected web-interface hosted by the University of Leicester, UK. Study participants will be assigned a study-specific identification code.

Ethics and dissemination

The study received ethical approvals from the University of Leicester research ethics committee (United Kingdom) (Ref No. 22349) and locally from the Mulago Hospital Research and Ethics Committee (MHREC1478), Kampala, Uganda as well as the Uganda National Council for Science and Technology (SS5105).

Confidentiality

The confidentiality of all participants will be protected to the fullest extent possible. All patient information will be kept secure and will be available only to the treatment staff and representatives of the sponsors, regulators, and ethics committees.

All participants will be provided with a unique identification number which will be recorded in the participant enrolment log and stored in a secure place. Study participants will not be

For peer review only

For peer review only - http://bmjopen.bmj.com/site/about/guidelines.xhtml

identified by name on any case report form, email or on any other documentation sent to the central database and will not be reported by name in any report, presentation or publication resulting from data collected in this study. Participants' data/specimens will be identified by study number or hospital number only.

Dissemination

Results of the study will be published in peer-reviewed journals and findings disseminated at appropriate local, regional and international scientific meetings and conferences. Social media will be used to disseminate information and summaries of results to a wider public domain. Furthermore, a participant dissemination meeting will be held following this trial, in which study participants will receive a summary of the findings.

COVID-19 provisions

Modifications will be made to the delivery of the PR program due to the Corona Virus Disease 2019 (COVID-19) pandemic. The PR room will be re-organized to allow for social distancing (minimum 2-meters) for both study staff and study participants. The maximum number of participants participating in the PR session will be reduced from 12 to 8 to ensure social distancing between participants. Before accessing the PR room, all participants and staff will be required to undergo temperature measurement using a hand-held non-contact thermometer, wash hands with soap or alcohol-based hand sanitizer. All participants will be provided with face masks during PR sessions. All surfaces inside the PR room will be disinfected before and after every PR session. PR sessions will be conducted in the morning hours to allow participants travel back home in time before the evening rush hour and the standard operating procedure for data collection will be modified ensure 2-meter distancing between the study staff and study participant. Study participants will undergo COVID-19 testing before starting PR and as needed during the hospital based sessions. All study staff will be required to wear N95 masks at all times and will undergo COVID-19 training with emphasis on infection prevention and control, and screening study participants for signs and symptoms of the disease.

Figure legend

Figure 1: Figure showing the study flow in the Post TB Pulmonary Rehabilitation trial

Legend*: PTBLD-post-TB lung disease; PR – Pulmonary Rehabilitation; R&A – Recruitment and Assessment*

For peer review only - http://bmjopen.bmj.com/site/about/guidelines.xhtml

Contributors

All authors have substantially contributed to the conception and design of the study. WK drafted the manuscript. All authors of the paper have revised the content and approved the final version to be published. All authors are accountable for all aspects of the work.

Competing interests

None declared

Funding

This research was funded by the National Institute for Health Research (NIHR) (17/63/20) using UK aid from the UK Government to support global health research. The views expressed in this publication are those of the author(s) and not necessarily those of the NIHR or the UK Department of Health and Social Care.

Data sharing

Following the completion of the project, data from the Global Health Research Group on Respiratory Rehabilitation (Global RECHARGE) Core Dataset [16] will be made available to the wider community upon reasonable request.

For peer review only - http://bmjopen.bmj.com/site/about/guidelines.xhtml

References

1. WHO, *Global tuberculosis report 2019.* 2019.
2. Dye C, H.A., Maher D, S. Hosseini SM, Nkhoma W, and Salaniponi FM., *Disease and Mortality in Sub-Saharan Africa. 2nd edition. Chapter 13. Tuberculosis*. 2006.
3. Pasipanodya, J.G., et al., *Pulmonary impairment after tuberculosis.* Chest, 2007. **131**(6): p. 1817-1824.
4. Harries, A.D., et al., *Successfully treated but not fit for purpose: paying attention to chronic lung impairment after TB treatment.* The International Journal of Tuberculosis and Lung Disease, 2016. **20**(8): p. 1010-1014.
5. van Kampen, S.C., et al., *International research and guidelines on post-tuberculosis chronic lung disorders: a systematic scoping review.* BMJ Global Health, 2018. **3**(4): p. e000745.
6. Chakaya, J., B. Kirenga, and H. Getahun, *Long term complications after completion of pulmonary tuberculosis treatment: A quest for a public health approach.* Journal of Clinical Tuberculosis and Other Mycobacterial Diseases, 2016. **3**: p. 10-12.
7. Nici, L., et al., *American thoracic society/European respiratory society statement on pulmonary rehabilitation.* American journal of respiratory and critical care medicine, 2006. **173**(12): p. 1390-1413.
8. Allwood, B., et al., *Post-tuberculosis lung health: perspectives from the First International Symposium.* 2020. **24**(8): p. 820-828.
9. Visca, D., et al., *Post-tuberculosis sequelae: the need to look beyond treatment outcome.* 2020. **24**(8): p. 761-762.
10. Lacasse, Y., et al., *Meta-analysis of respiratory rehabilitation in chronic obstructive pulmonary disease. A Cochrane systematic review*. 2007.
11. McCarthy, B., et al., *Pulmonary rehabilitation for chronic obstructive pulmonary disease.* Cochrane database of systematic reviews, 2015(2).
12. Bolton, C.E., et al., *British Thoracic Society guideline on pulmonary rehabilitation in adults: accredited by NICE.* Thorax, 2013. **68**(Suppl 2): p. ii1-ii30.
13. Spruit, M.A., et al., *An official American Thoracic Society/European Respiratory Society statement: key concepts and advances in pulmonary rehabilitation.* American journal of respiratory and critical care medicine, 2013. **188**(8): p. e13-e64.
14. Jones, R., et al., *A pre–post intervention study of pulmonary rehabilitation for adults with post-tuberculosis lung disease in Uganda.* International Journal of Chronic Obstructive Pulmonary Disease, 2017. **12**: p. 3533.
15. Jones, R., et al., *A development study of pulmonary rehabilitation for patients with chronic lung disease in Uganda*. 2016, Eur Respiratory Soc.
16. Orme, M.W., M. Orme, and R.J.J.o.G.H. Free, *Global RECHARGE: Establishing a standard international data set for pulmonary rehabilitation in low-and middle-income countries.* 2020. **10**(2).
17. Graham, B.L., et al., *Standardization of spirometry 2019 update. An official American thoracic society and European respiratory society technical statement.* 2019. **200**(8): p. e70-e88.
18. Evans, R.A. and S.J.J.T. Singh, *Minimum important difference of the incremental shuttle walk test distance in patients with COPD.* 2019. **74**(10): p. 994-995.
19. Powers, S.K., S.L. Dodd, and E.M. Jackson, *Total fitness & wellness*. 2013: Pearson Higher Ed.
20. Borg, G., *Perceived exertion as an indicator of somatic stress.* Scandinavian journal of rehabilitation medicine, 1970.
21. Borg, G.A., *Psychophysical bases of perceived exertion.* Medicine & science in sports & exercise, 1982.
22. Revill, S., et al., *The endurance shuttle walk: a new field test for the assessment of endurance capacity in chronic obstructive pulmonary disease.* Thorax, 1999. **54**(3): p. 213-222.

For peer review only - http://bmjopen.bmj.com/site/about/guidelines.xhtml

23. Singh, S.J., et al., *Development of a shuttle walking test of disability in patients with chronic airways obstruction.* Thorax, 1992. **47**(12): p. 1019-1024.
24. Van der Molen, T., et al., *Development, validity and responsiveness of the Clinical COPD Questionnaire.* Health and quality of life outcomes, 2003. **1**(1): p. 13.
25. Kon, S.S., et al., *The Clinical COPD Questionnaire: response to pulmonary rehabilitation and minimal clinically important difference.* Thorax, 2014. **69**(9): p. 793-798.
26. Jones, P., et al., *Development and first validation of the COPD Assessment Test.* European Respiratory Journal, 2009. **34**(3): p. 648-654.
27. Kon, S.S., et al., *Minimum clinically important difference for the COPD Assessment Test: a prospective analysis.* The Lancet Respiratory Medicine, 2014. **2**(3): p. 195-203.
28. Nowak, C., et al., *Accuracy of the hospital anxiety and depression scale for identifying depression in chronic obstructive pulmonary disease patients.* Pulmonary medicine, 2014. **2014**.
29. Smid, D.E., et al., *Responsiveness and MCID estimates for CAT, CCQ, and HADS in patients with COPD undergoing pulmonary rehabilitation: a prospective analysis.* Journal of the American Medical Directors Association, 2017. **18**(1): p. 53-58.
30. Wynne, S., et al., *The Hospital Anxiety and Depression Scale (HADS) in Bronchiectasis: Response to pulmonary rehabilitation (PR) and Minimum Clinically Important Difference (MCID)*. 2019, Eur Respiratory Soc.
31. Kroenke, K. and R.L. Spitzer, *The PHQ-9: a new depression diagnostic and severity measure.* Psychiatric annals, 2002. **32**(9): p. 509-515.
32. Löwe, B., et al., *Monitoring depression treatment outcomes with the patient health questionnaire-9.* Medical care, 2004: p. 1194-1201.
33. Reilly, M.C., A.S. Zbrozek, and E.M. Dukes, *The validity and reproducibility of a work productivity and activity impairment instrument.* Pharmacoeconomics, 1993. **4**(5): p. 353-365.
34. Group, T.E., *EuroQol-a new facility for the measurement of health-related quality of life.* Health policy, 1990. **16**(3): p. 199-208.
35. Nolan, C.M., et al., *The EQ-5D-5L health status questionnaire in COPD: validity, responsiveness and minimum important difference.* Thorax, 2016. **71**(6): p. 493-500.
36. Bohannon, R.W., *Sit-to-stand test for measuring performance of lower extremity muscles.* Perceptual and motor skills, 1995. **80**(1): p. 163-166.
37. Jones, S.E., et al., *The five-repetition sit-to-stand test as a functional outcome measure in COPD.* Thorax, 2013. **68**(11): p. 1015-1020.
38. Stenton, C., *The MRC breathlessness scale.* Occupational Medicine, 2008. **58**(3): p. 226-227.
39. De Torres, J.P., et al., *Power of outcome measurements to detect clinically significant changes in pulmonary rehabilitation of patients with COPD.* Chest, 2002. **121**(4): p. 1092-1098.
40. Crisafulli, E. and E.M. Clini, *Measures of dyspnea in pulmonary rehabilitation.* Multidisciplinary respiratory medicine, 2010. **5**(3): p. 202.
41. Zatloukal, J., et al., *The minimal important difference for the endurance shuttle walk test in individuals with chronic obstructive pulmonary disease following a course of pulmonary rehabilitation.* Chronic Respiratory Disease, 2019. **16**: p. 1479973119853828.
42. Bassett, D.R. and D. John, *Use of pedometers and accelerometers in clinical populations: validity and reliability issues.* Physical therapy reviews, 2010. **15**(3): p. 135-142.
43. Lucas, A.O. and H.M. Gilles, *Short textbook of public health medicine for the tropics. Chapter-Approaches to economic evaluation*. 2003: CRC Press.
44. Harris, P.A., et al., *Research electronic data capture (REDCap)—a metadata-driven methodology and workflow process for providing translational research informatics support.* Journal of biomedical informatics, 2009. **42**(2): p. 377-381.

For peer review only - http://bmjopen.bmj.com/site/about/guidelines.xhtml

45. Harris, P.A., et al., *The REDCap consortium: Building an international community of software platform partners.* Journal of biomedical informatics, 2019. **95**: p. 103208.

For peer review only

For peer review only - http://bmjopen.bmj.com/site/about/guidelines.xhtml

Table 1: The table shows the assessment and follow up schedule

Observation/Investigation	Screening/ Baseline assessments	Randomisation	12 weeks of study participation	
			Hospital based Pulmonary Rehab	Follow-up phase of homebased exercises
			End of 6 weeks of PR	End of 6 weeks of home exercises
Written informed consent	x	x		
Demographics	x	x		
Medical history	x	x		
Clinical exam	x	x		
Chest X-ray	x			
Spirometry		x		
MRC dyspnoea grade	x		x	x
Assess symptoms	x		x	x
Incremental Shuttle Walk Test	x		x	x
Endurance Shuttle Walk Test	x		x	x
Borg breathlessness scale	x		x	x
Mid Upper Arm Circumference	x		x	x
Sit-to-stand time	x		x	x
COPD Assessment Test		x	x	x
Clinical COPD Questionnaire		x	x	x
Patient Health Questionnaire		x	x	x
HADS		x	x	x
WPAI		x	x	x
Physical Activity (Actigraph monitor)		x	x	x
Cost/Benefit Analysis		x	x	x
EQ-5D-5L Questionnaire		x	x	x

For peer review only

For peer review only

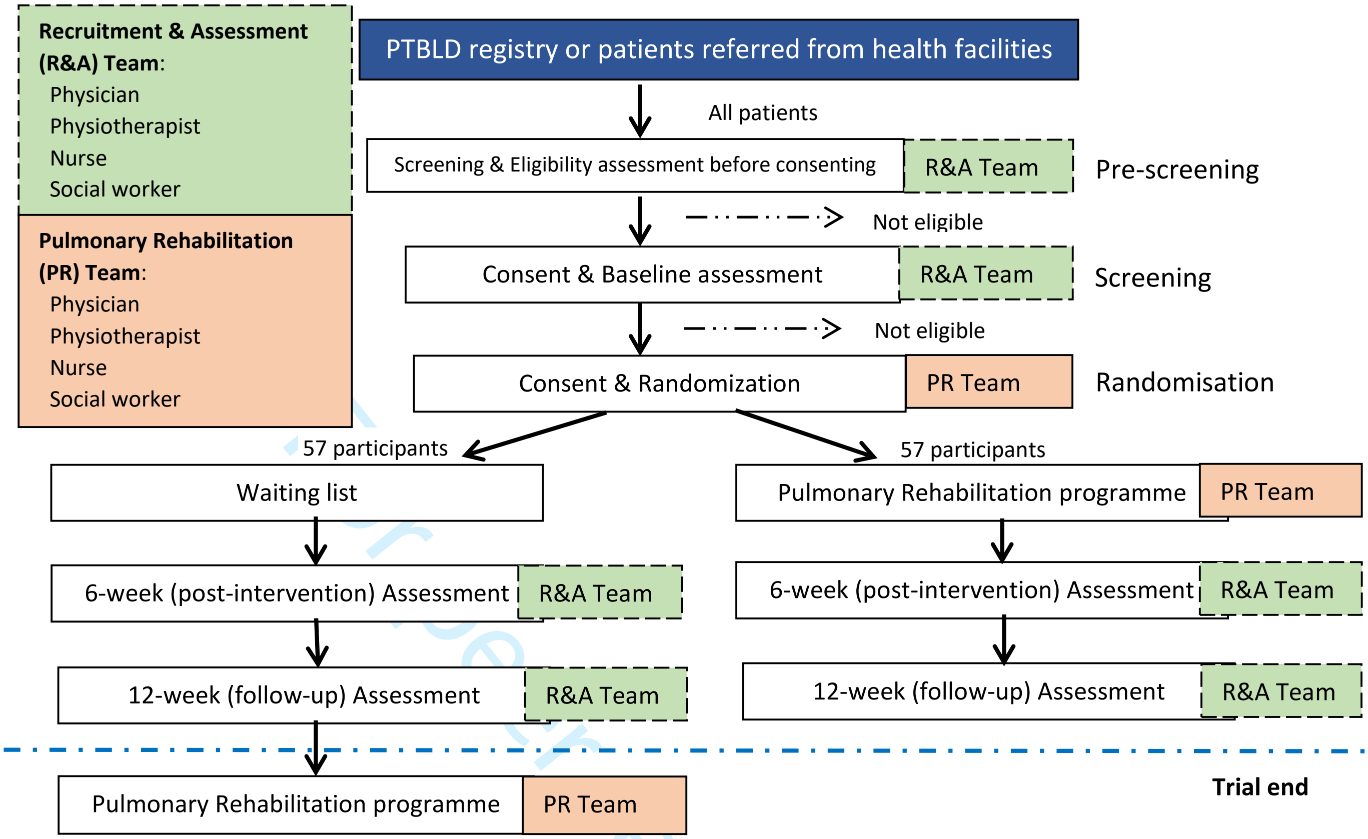

Figure 1: Figure showing the study flow in the Post TB Pulmonary Rehabilitation trial

***Legend**: PTBLD-post-TB lung disease; PR – Pulmonary Rehabilitation; R&A – Recruitment and Assessment*

For peer review only

Supplementary Table 1: Physical activity data collection and accelerometry processing criteria

Criteria	Details
**Accelerometer Model**	ActiGraph wGT3X-BT (version 6.13.4; firmware 1.9.2)
**Serial number range**	Twenty unique devices will be used; ranging from MOS2E09190617 to MOS2E25190750 and averaging six deployments per device (same serial used for baseline and follow-up wear periods to remove any inter-device variability)
**Piezosensor orientation**	Triaxial
**Mode setup**	Mode 29 (x, y, z, steps, lux)
**Original sample rate**	100 Hz (.gt3x file format)
**Deployment method**	Baseline:<br>Fitted by research team on Day 0 (Baseline PR Assessment)<br>Fitted by participant on Day 1<br>Follow-up:<br>Fitted by research team Day 0 (11th/12th session)<br>Fitted by participant on Day 1
**Location worn**	Anterior hip adjacent to the mid-line of the thigh
**Requested days of wear**	7 days of free-living (10,080 epochs)
**Initialization**	Not deployed in delay mode in order to standardised capture of Day 0 (00:00) with stop time based on date of first PR class (baseline) and date of follow-up assessment
**Wear instructions**	Wear continuously except for sleep and water-based activities
**Non-wear appropriation**	≥60 min of consecutive 0s with allowance for 2 minutes of interruptions
**Valid day criteria**	≥8 hours of valid waking wear time
**Valid file**	≥4 valid days for each of the two time points
**Missing data**	Data modelling or imputation will not be performed
**Epoch length**	60 seconds
**Intensity classification (absolute)**	Uniaxial (x-axis) intensity cut-points as follows: Stationary <100 cpm; Light 100-2019 cpm; Moderate 2020-5998cpm; Vigorous ≥5999cpm (Moderate-to-vigorous ≥2020 cpm)
**Intensity classification (relative)**	Uniaxial (x-axis) cut-points based on Endurance Shuttle Walk Test performance

PR – Pulmonary Rehabilitation

Supplementary Table 2: A list of warm up and cool down activities during PR session
1. Marching on the spot, slowly bringing the feet off the floor for up to 1 minute<br>2. Heel digs: alternate heel digs in front of the body with toes pointing to the ceiling, add in a biceps curl (repeat 10-12 times).<br>3. Toe taps: Tap the toes to the floor in front of alternating legs at a comfortable distance. Heels stay off the ground (repeat 10-12 times).<br>4. High knee marching – with opposite hand to opposite knee.<br>5. Side bends: With arms relaxed by your side, leaning over to the right for 8 to 10 seconds and back to centre, then lean to the left for 8 to 10 seconds and back to the centre (repeat 8 times).<br>6. Arms stretched up, forwards and down.<br>7. Alternate punching of arms forward.<br>8. Seated or standing side taps to the floor with the foot – alternate legs.<br>9. Seated or standing in upright posture, feet placed shoulder breadth apart – shoulder roll in both directions (clockwise and anti-clockwise).<br>10. Seated or standing in upright posture, feet placed shoulder breath apart, elbows bent with hands onto shoulder – elbows make circles in clockwise and anti-clockwise.<br>11. Hamstring stretch: With right leg straight, place it in front of the body, heel pushed into the floor with toes pointing toward the ceiling. Slightly bend the left knee, place hands on the straight right leg and gently lean forward. Hold the stretch for 10-15 seconds then return to upright position. Repeat on left leg.<br>12. Quadriceps stretch: While holding a chair or onto a wall, stand on your left leg and grab your right foot using your right hand, pulling it gently towards the ceiling. Hold the position for 10-15 seconds and return to upright position and repeat on the right leg.

PR – Pulmonary Rehabilitation

CONSORT 2010 checklist of information to include when reporting a randomised trial*

Section/Topic	Item No	Checklist item	Reported on page No
**Title and abstract**			
	1a	Identification as a randomised trial in the title	1
	1b	Structured summary of trial design, methods, results, and conclusions (for specific guidance see CONSORT for abstracts)	2
**Introduction**			
Background and objectives	2a	Scientific background and explanation of rationale	3,4
	2b	Specific objectives or hypotheses	4
**Methods**			
Trial design	3a	Description of trial design (such as parallel, factorial) including allocation ratio	4,5
	3b	Important changes to methods after trial commencement (such as eligibility criteria), with reasons	15
Participants	4a	Eligibility criteria for participants	5,6
	4b	Settings and locations where the data were collected	5
Interventions	5	The interventions for each group with sufficient details to allow replication, including how and when they were actually administered	6,7,8
Outcomes	6a	Completely defined pre-specified primary and secondary outcome measures, including how and when they were assessed	9,10,11,12
	6b	Any changes to trial outcomes after the trial commenced, with reasons	
Sample size	7a	How sample size was determined	7
	7b	When applicable, explanation of any interim analyses and stopping guidelines	
Randomisation:			
Sequence generation	8a	Method used to generate the random allocation sequence	6
	8b	Type of randomisation; details of any restriction (such as blocking and block size)	
Allocation concealment mechanism	9	Mechanism used to implement the random allocation sequence (such as sequentially numbered containers), describing any steps taken to conceal the sequence until interventions were assigned	6
Implementation	10	Who generated the random allocation sequence, who enrolled participants, and who assigned participants to interventions	
Blinding	11a	If done, who was blinded after assignment to interventions (for example, participants, care providers, those	7,8

For peer review only

		assessing outcomes) and how	
	11b	If relevant, description of the similarity of interventions	
Statistical methods	12a	Statistical methods used to compare groups for primary and secondary outcomes	13,14
	12b	Methods for additional analyses, such as subgroup analyses and adjusted analyses	
**Results**			
Participant flow (a diagram is strongly recommended)	13a	For each group, the numbers of participants who were randomly assigned, received intended treatment, and were analysed for the primary outcome	
	13b	For each group, losses and exclusions after randomisation, together with reasons	
Recruitment	14a	Dates defining the periods of recruitment and follow-up	
	14b	Why the trial ended or was stopped	
Baseline data	15	A table showing baseline demographic and clinical characteristics for each group	
Numbers analysed	16	For each group, number of participants (denominator) included in each analysis and whether the analysis was by original assigned groups	
Outcomes and estimation	17a	For each primary and secondary outcome, results for each group, and the estimated effect size and its precision (such as 95% confidence interval)	
	17b	For binary outcomes, presentation of both absolute and relative effect sizes is recommended	
Ancillary analyses	18	Results of any other analyses performed, including subgroup analyses and adjusted analyses, distinguishing pre-specified from exploratory	
Harms	19	All important harms or unintended effects in each group (for specific guidance see CONSORT for harms)	
**Discussion**			
Limitations	20	Trial limitations, addressing sources of potential bias, imprecision, and, if relevant, multiplicity of analyses	
Generalisability	21	Generalisability (external validity, applicability) of the trial findings	
Interpretation	22	Interpretation consistent with results, balancing benefits and harms, and considering other relevant evidence	
**Other information**			
Registration	23	Registration number and name of trial registry	3
Protocol	24	Where the full trial protocol can be accessed, if available	
Funding	25	Sources of funding and other support (such as supply of drugs), role of funders	1,16

*We strongly recommend reading this statement in conjunction with the CONSORT 2010 Explanation and Elaboration for important clarifications on all the items. If relevant, we also recommend reading CONSORT extensions for cluster randomised trials, non-inferiority and equivalence trials, non-pharmacological treatments, herbal interventions, and pragmatic trials. Additional extensions are forthcoming: for those and for up to date references relevant to this checklist, see www.consort-statement.org.

For peer review only - http://bmjopen.bmj.com/site/about/guidelines.xhtml

BMJ Open

A study protocol for a randomised controlled trial assessing the impact of Pulmonary Rehabilitation on maximal exercise capacity for adults living with Post-TB lung disease: Global RECHARGE Uganda

Journal:	*BMJ Open*
Manuscript ID	bmjopen-2020-047641.R1
Article Type:	Protocol
Date Submitted by the Author:	18-Apr-2021

Complete List of Authors:	Katagira, Winceslaus; Makerere University, Lung Institute<br>Orme, Mark; University of Leicester, Respiratory Sciences<br>Jones, Amy; University of Leicester, Respiratory Sciences<br>Kasiita, Richard; Mulago Hospital, Kampala, Uganda, Department of Physiotherapy,<br>Rupert, Jones; University of Plymouth (UK), Faculty of Health<br>Barton, Andy; University of Plymouth (UK), Faculty of Health<br>Miah, Ruhme ; University of Leicester, Department of Respiratory Sciences<br>Manise, Adrian ; University of Leicester, Department of Respiratory Sciences<br>Matheson, Jesse; University of Sheffield, Department of Economics<br>Free, Robert; Centre for Exercise and Rehabilitation Science (CERS), NIHR Leicester Biomedical Research Centre – Respiratory, University Hospitals of Leicester NHS Trust<br>Steiner, michael ; Institute for Lung Health, NIHR Leicester Biomedical Research Centre – Respiratory, University of Leicester, Glenfield Hospital,<br>Kirenga, Bruce; Makerere University, Lung Institute<br>Singh, Sally; University Hospitals of Leicester NHS Trust, Cardiac/Pulmonary Rehabilitation

**Primary Subject Heading**:	Respiratory medicine
Secondary Subject Heading:	Rehabilitation medicine
Keywords:	Rehabilitation medicine < INTERNAL MEDICINE, Health economics < HEALTH SERVICES ADMINISTRATION & MANAGEMENT, Tuberculosis < INFECTIOUS DISEASES, Adult thoracic medicine < THORACIC MEDICINE

SCHOLARONE™
Manuscripts

I, the Submitting Author has the right to grant and does grant on behalf of all authors of the Work (as defined in the below author licence), an exclusive licence and/or a non-exclusive licence for contributions from authors who are: i) UK Crown employees; ii) where BMJ has agreed a CC-BY licence shall apply, and/or iii) in accordance with the terms applicable for US Federal Government officers or employees acting as part of their official duties; on a worldwide, perpetual, irrevocable, royalty-free basis to BMJ Publishing Group Ltd ("BMJ") its licensees and where the relevant Journal is co-owned by BMJ to the co-owners of the Journal, to publish the Work in this journal and any other BMJ products and to exploit all rights, as set out in our licence.

The Submitting Author accepts and understands that any supply made under these terms is made by BMJ to the Submitting Author unless you are acting as an employee on behalf of your employer or a postgraduate student of an affiliated institution which is paying any applicable article publishing charge ("APC") for Open Access articles. Where the Submitting Author wishes to make the Work available on an Open Access basis (and intends to pay the relevant APC), the terms of reuse of such Open Access shall be governed by a Creative Commons licence – details of these licences and which Creative Commons licence will apply to this Work are set out in our licence referred to above.

Other than as permitted in any relevant BMJ Author's Self Archiving Policies, I confirm this Work has not been accepted for publication elsewhere, is not being considered for publication elsewhere and does not duplicate material already published. I confirm all authors consent to publication of this Work and authorise the granting of this licence.

For peer review only

Title: A study protocol for a randomised controlled trial assessing the impact of Pulmonary Rehabilitation on maximal exercise capacity for adults living with Post-TB lung disease: Global RECHARGE Uganda

Authors

Winceslaus Katagira[1], Mark W. Orme[2,3], Amy V. Jones[2,3], Richard Kasiita[4], Rupert Jones[5], Andy Barton[5], Ruhme Miah[2,3], Adrian Manise[6], Jesse Matheson[7], Robert C. Free[3], Michael C. Steiner[2,3], Bruce Kirenga[1] and Sally J. Singh[2,3]

Affiliations

1. Makerere University Lung Institute, Kampala, Uganda
2. Department of Respiratory Sciences, University of Leicester (UK)
3. Centre for Exercise and Rehabilitation Science (CERS), NIHR Leicester Biomedical Research Centre – Respiratory, University Hospitals of Leicester NHS Trust - Leicester (UK)
4. Department of Physiotherapy, Mulago Hospital, Kampala, Uganda
5. Faculty of Health, University of Plymouth (UK)
6. NIHR Leicester Biomedical Research Centre, University Hospitals of Leicester NHS Trust - Leicester (UK)
7. Department of Economics, University of Sheffield, Sheffield, United Kingdom

Protocol version:

Version 1.0 July 2019

Funding source:

This research was funded by the National Institute for Health Research (NIHR) (17/63/20) using UK aid from the UK Government to support global health research. The views expressed in this publication are those of the author(s) and not necessarily those of the NIHR or the UK Department of Health and Social Care.

Roles and responsibilities of authors

a) Substantially contributed to the conception and/ or design of the work
b) Substantially contributed to the acquisition, analysis, or interpretation of data for the work
c) Substantially contributed to the drafting of the work and/ or revising it critically for important intellectual content

Name and contact information for corresponding author

Katagira Winceslaus, wincegira@gmail.com

Word count: 3,998

For peer review only - http://bmjopen.bmj.com/site/about/guidelines.xhtml

Abstract

Introduction

The burden of post-TB lung disease (PTBLD) is steadily increasing in sub-Saharan Africa, causing disability among TB survivors. Without effective medicines, the mainstay of PTBLD treatment evolves around disease prevention and supportive treatment. Pulmonary rehabilitation (PR), a low-cost, non-pharmacological intervention has shown effectiveness in a group of PTBLD individuals but has not been tested in a clinical trial. This study aims to assess the impact of a 6-week PR programme on maximal exercise capacity and other outcomes among adults in Uganda living with PTBLD.

Methods and analysis

This is a randomized waiting-list controlled trial with blinded outcome measures, comparing PR versus usual care for patients with PTBLD. A total of 114 participants will be randomized (1:1) to receive either usual care (on the waiting list) or PR, with follow-up assessments at 6- and 12-weeks post-intervention. The primary outcome is change in walking distance measured by the Incremental Shuttle Walk Test from baseline to the end of 6-weeks of PR. All secondary outcomes will be compared between the PR and usual care arms from baseline to 6- and 12-week follow-ups. Secondary outcomes include self-reported respiratory symptoms, physical activity, psychological well-being, health related quality of life and cost benefit analysis. All randomized participants will be included in the intention to treat analysis population. The primary efficacy analysis will be based on both per protocol and modified intention-to-treat populations.

Ethics and dissemination

The trial has received ethical clearance from the Mulago Hospital Research and Ethics Committee (MHREC 1478), Kampala, Uganda as well as the Uganda National Council for Science and Technology (SS 5105). Ethical approval has been obtained from the University of Leicester, United Kingdom research ethics committee (Ref No. 22349). Study findings will be published in appropriate peer-reviewed journals and disseminated at appropriate local, regional and international scientific meetings and conferences.

Strengths and limitations of this study

The study aims to determine the effectiveness of PR for individuals with PTBLD in a clinical trial setting. To our knowledge, this is the first pragmatic, fully powered effectiveness trial for

For peer review only

PR in PTBLD in Africa. This is a progression of previous work that established feasibility and acceptability of PR design for people living with PTBLD in Uganda.

Due to funding limitations, we are unable to carry out a multi-site study. This may limit generalizability of the study findings.

Trial registration: ISRCTN18256843

Key words

Pulmonary Rehabilitation, Post-TB lung disease, Chronic Respiratory disease, non-drug treatment, Exercise, Education

Introduction

Background and rationale

In 2018, 24% of the global Tuberculosis (TB) incident cases occurred in the African region [1]. Furthermore, 24 of the 30 high TB/Human Immunodeficiency Virus (HIV) burden countries, including Uganda, are in the African region; accounting for 71% of the global burden of HIV associated TB [1]. Despite great strides made over the recent years to achieve the 90% treatment success rate, as part of the "End TB strategy" target [1], a significant number of TB survivors continue to have poor health related quality of life [2]. This may be attributed to the pulmonary function impairment following TB treatment which has been reported in approximately 50% of pulmonary TB survivors [3]. The reduction in ventilation and perfusion attributed to the permanent lung parenchymal damage [4] clinically manifests as long-term respiratory symptoms and eventually chronic respiratory disease (CRD), including chronic obstructive pulmonary disease (COPD), bronchiectasis and aspergillosis [5, 6].

Adults with post-TB respiratory symptoms develop skeletal muscle dysfunction, related to physical inactivity and systemic inflammation, which is often compounded by impaired nutrition and poverty [7]. Such patients enter a vicious cycle with falling body weight, progressive morbidity and increased mortality [7]. Individuals affected by CRDs tend to avoid exercise and become increasingly deconditioned and demotivated, leading to a cycle of decline. There are no effective medicines for post-TB lung disease (PTBLD) and the mainstay of

For peer review only - http://bmjopen.bmj.com/site/about/guidelines.xhtml

treatment evolves around disease prevention and supportive treatment. The disease, previously neglected by health services and researchers, is now the focus of increasing interest [8, 9].

In low- and middle-income countries (LMIC) where health care focuses on treatment and prevention of infectious diseases, as opposed to managing chronic diseases, the care for adults living with CRD presents a major challenge. Consequently, patients that require long-term and systemic approaches often receive sub-optimal medical care, inevitably leading to preventable deaths in resource poor settings.

Pulmonary Rehabilitation (PR) is a low cost, high impact intervention that reverses the disability associated with CRDs, and is supported by the highest level of research evidence in high income countries [10, 11]. A PR programme brings together health professionals from many disciplines offering supervised exercise training and disease education, supporting people to manage their own disease. However, in LMIC where the burden of CRDs is increasing fastest, PR is scarce and health care services are poorly adapted to deal with such diseases. Although PR is a grade "A" evidence treatment for adults with COPD [12] and has been utilized in other chronic lung diseases [13], it's efficacy in Post TB lung disease is not known. In a development study to examine the impact of PR for people with PTBLD in Uganda, it was feasible to run a PR programme and participants reported clinically important improvements in quality of life, exercise capacity, and respiratory outcomes [14]. To date, there has been little attention to the role of PR in PTBLD globally, particularly in Africa where a significant number of PTB survivors reside.

Study Objectives

The primary objective of this trial is to assess the impact of a 6-week PR programme on maximal exercise capacity using the incremental shuttle walking test (ISWT) among adults living with PTBLD post-intervention.

The secondary objectives include assessing the impact of PR on quality of life and other outcomes for patients with Post-TB lung disease, and to conduct a cost-benefit analysis of PR.

Methods

Study design

This is a prospective, randomised waiting-list controlled trial with blinded outcome measures, comparing PR versus usual care for patients with post-TB lung disease. During this effectiveness trial, a total of 114 participants will be randomized (1:1) to receive either usual care (waiting-list) or PR (Figure 1).

For peer review only - http://bmjopen.bmj.com/site/about/guidelines.xhtml

Study setting

The study is conducted at the PR centre located at the Makerere University Lung Institute (MLI) Clinic, Kampala, Uganda. The MLI clinic is an academic outpatient clinic within the Mulago National Referral hospital, a teaching and clinical research hospital for Makerere University.

Study population

Recruitment

Adults with PTBLD will be referred from health facilities and clinics (TB treatment centres and HIV/TB caring centres) around Kampala to the PR centre. Existing registers have around 300 adults living with PTBLD and additional patients will be screened directly from the outpatient departments.

In this study, a patient is considered to have post-TB lung disease (PTBLD) if they successfully completed treatment for microbiologically confirmed Pulmonary TB but continue to experience chronic respiratory symptoms with radiological evidence of lung parenchymal damage.

Participant invitation

The process of identifying and inviting eligible patients was refined in the development study. Eligible individuals identified as having an established PTBLD diagnosis will be received at the PR centre at the MLI. Literate participants will be asked to read the patient information sheet (PIS) about the study, written in English or translated in the local language. Illiterate participants will have the contents read to them in full by a study staff, in the presence of a witness who will be present during the whole process. Participants will have the opportunity to discuss the PIS with the study medical personnel. Once the study staff are satisfied that the participant has understood the PIS, and is interested in taking part in the study, they will be taken through the informed consent process. Participants will give consent before undergoing screening tests and procedures, and if still eligible after the screening process, will be taken through another informed consent process for randomisation.

Eligibility criteria

Inclusion criteria

A patient with PTBLD is eligible for the trial if they meet all of the following criteria: aged ≥18 years, willing and able to provide written informed consent (signed or witnessed consent if the patient is illiterate), a documented past history of smear positive pulmonary TB with

For peer review only

treatment completed ≥6 months prior to study enrolment, a negative Xpert MTB/RIF assay for *Mycobacterium tuberculosis* at the time of study enrolment, and report a Medical Research Council (MRC) dyspnoea grade ≥2.

Exclusion criteria

A PTBLD patient is ineligible for the study if they have co-morbidities that preclude exercise (e.g. known unstable cardiovascular disease, locomotor difficulties) or if they are unwilling to participate for any reason or had any condition (social or medical) which in the opinion of the investigator would make study participation unsafe.

Randomization

Once eligible participants have consented to take part in the study, they will be randomised using a web-based randomisation system (https://www.sealedenvelope.com/). Participants will be randomized (1:1) to receive either usual care or PR. Access to the web-based system will be controlled through an authorised username and password. Randomisations will be conducted by a member of the study team independent from the data collection team and will be revealed to the data collection and intervention delivery teams after baseline measurements have been obtained.

Participant timeline

After randomisation, the PR team will explain to participants when the PR sessions will take place. For each individual participant, the hospital based PR programme will last six weeks followed by a follow-up period of six weeks of home exercises. Participants in the control arm (waiting-list) of the trial will be informed of the date for their first exercise session in approximately 12-15 weeks. Based on our development study [15], we expect to find prolonged and possibly improved effects of PR at follow-up. Our experience indicates that a follow-up period of more than three months after the start of the PR programme would be unrealistic in this environment without unacceptable attrition. Study participants will receive compensation for their time and transport.

Pulmonary Rehabilitation Team

The PR team has received adequate training on the delivery of PR and participated in the development study which informed this trial [14]. Furthermore, the individuals are registered health professionals (physiotherapist, physicians, and nurses) and have undertaken training regarding the study tests, procedures and measurements per protocol as well as Good Clinical Practice.

For peer review only - http://bmjopen.bmj.com/site/about/guidelines.xhtml

Assessment and follow up

Participants in both arms of the trial will be asked to attend the baseline, 6-week and 12-week post-intervention assessment visits at the PR centre at MLI. Data will be collected by the study staff (medical doctor, nurse and physiotherapist). Table 1 shows all baseline and follow up assessment data that will be collected during the trial, in accordance with a minimum recommended dataset for PR trials in LMIC[16].

Study procedures

During the screening visit, prospective participants will undergo clinical examination, MRC dyspnoea grading, sputum examination using Xpert MTB/RIF assay and a frontal chest radiograph. In addition, demographic, socio-economic, medical and clinical history (including respiratory symptoms and exposure history to cigarettes and biomass) will be collected using a standardised questionnaire. At the randomisation visit, spirometry will be performed using American Thoracic Society and European Respiratory Society guidelines [17].

Sample size

The study will be powered to detect a 35m difference in the ISWT measured at baseline and after completion of PR[18]. Assuming that ISWT follows an approximately normal distribution, a power calculation based on a paired t-test was performed. Based on a trial sample size of 40 participants in each of the treatment and control groups, a 2-sided 5% significance level and a statistical power of 80%, the clinically important change in ISWT of 35m will also be statistically significant. Our recent feasibility study [15] was used to obtain an estimate of the pooled standard deviation for the power calculation. Conservatively assuming up to 30% loss to follow-up at 6-weeks, a total of 114 participants are required to be recruited and randomised (1:1) to each arm (PR: 57 participants or waiting list: 57 participants). Using the 70% ineligibility rate during screening from the feasibility study, we will need to screen approximately 543 PTBLD patients.

Blinding (masking)

Due to the nature of PR, it will not be possible to blind participants to their group allocation but participants will be asked not to reveal their group during the follow-up assessments. The participant and treating clinician will be aware of treatment allocation, however, the outcome measures will be performed by staff blinded to treatment allocation and the ISWT (primary outcome) will be prioritised to reduce the risk of un-blinding. Any episodes of un-blinding will be documented and reported.

For peer review only - http://bmjopen.bmj.com/site/about/guidelines.xhtml

Treatment arms

Usual care (control arm)

The participants in the waiting-list (control) arm will receive usual care and will be offered PR after completing 12-weeks of follow-up. There are currently no guidelines for the clinical management of PTBLD both locally and internationally. Usual care will be optimised where possible and will include the following: frontal chest radiograph, spirometry to screen for airway diseases, inhalational therapies for airway disease amenable to treatment (where appropriate), antibiotic and systemic glucocorticoid therapy for infective exacerbations (where appropriate), and verbal advice to quit smoking and reduce exposure to biomass smoke. According to local practice, all post-TB patients with significant post-bronchodilator response on Spirometry (at least 12% and 200mls increase in forced expiratory volume in 1 second (FEV1)) are managed with a combination of inhaled corticosteroids and long-acting beta-agonists, while those with fixed airflow obstruction (post-bronchodilator FEV1/forced vital capacity (FVC) ratio of less than 0.70) are managed with long acting bronchodilators. PR will be offered as an adjunctive non-pharmacological treatment as recommended by international guidelines [19].

Pulmonary Rehabilitation (trial intervention arm)

In addition to usual care described above, participants in the intervention arm will receive PR. PR will consist of a six-week programme offered to a group of up to 12 participants, with sessions occurring twice weekly for at least two hours (approximately one hour for education and one hour for exercise).

Warm-up and cool-down

Before starting exercises, participants will be taken through a group warm up session, followed by a cool down session at the end of exercises, each lasting 10-15 minutes. Warm up is aimed at readying the body for both the physical aspects of performance (increased blood flow and muscle temperature) and mental readiness for exercise whilst cool down session facilitates a smoother decline in temperature and blood flow [20] Both warm up and cool down will consist of stretching and flexibility exercises during which participants will perform both upper and lower body flexibility exercises, held for 10 to 15 seconds each (including stretching of major muscle groups such as the calves, hamstrings, quadriceps, and biceps, as well as range of motion exercises for the neck, shoulders, and trunk), 2 days/week[13]. The cool down session has the same activities of warm-up (supplementary table 1) but performed at a slower pace.

For peer review only - http://bmjopen.bmj.com/site/about/guidelines.xhtml

For peer review only

Endurance training

Each participant will go through two stations of endurance exercise; load-adjustable stationary cycling and ground-based walking stations. We shall employ an intensity of continuous exercise at each station for 10 minutes or until a Borg dyspnoea score of 4-6 (moderate to [very] severe) is attained [21, 22]. Participants who may have difficulty in sustaining continuous high-intensity exercise will have interspersed periods of rest or lower intensity exercise to maximise the benefits of exercise training [13]. The walking exercise regime will be individually prescribed to participants based around their performance in the ISWT. Participants will be encouraged to walk at 85% of their maximal ISWT walking speed [23].

Strength training

Each participant will go through two stations for strengthening upper limb muscles (pull-ups and biceps curls) and two for strengthening lower limb muscles (sit-to-stand and step-up exercises). Each of the stations will include 3 sets of 8-12 repetitions. Participants will be asked to continue doing both endurance and resistance exercises at home, unsupervised.

Education sessions

A dedicated education session will be conducted at the start of each class, before the exercise regimes (Table 2; 12 sessions in total).

**Table 2: Education content of the Global RECHARGE Pulmonary Rehabilitation programme**
1. Normal anatomy and physiology of the lungs
2. Pathophysiology of chronic lung disease
3. Tuberculosis and how it causes lung damage
4. Coping with chronic lung disease and coping with stress
5. Avoidance of risk factors for chronic lung disease
6. Early recognition and treatment of exacerbations
7. Strategies for managing breathlessness
8. Energy conservation during activities of daily living
9. Role and rationale for medications and devices
10. Benefit of exercise and physical activities
11. Healthy food intake
12. Secretion clearance techniques

For peer review only

For peer review only - http://bmjopen.bmj.com/site/about/guidelines.xhtml

Study Outcomes

Primary outcome

The primary outcome is change in walking distance measured by the ISWT from pre to post-intervention. A group change of at least 35m is considered clinically important [18].

Incremental Shuttle Walking Test

The ISWT is frequently used as an outcome measure for PR [24]. Improvement in walking distance of 35m during the post-PR shuttle test, measured from baseline (pre-PR) using the ISWT is considered a clinically important difference [18]. The ISWT requires the patient to walk up and down a 10-meter course, identified by two cones inset 0.5m from either end to avoid the need for abrupt changes in direction. The speed at which the patient walks is dictated by an audio signal played on an audio device. Each participant will receive standardised instructions to: "Walk at a steady pace, aiming to turn around when you hear the signal. You should continue to walk until you feel that you are unable to maintain the required speed without becoming unduly breathless" [25]. To ensure the learning effect is accounted for, a practice ISWT will be performed and the participant will receive encouragement from the physiotherapist throughout the test in an effort to increase the distance one can walk. The test is terminated when either 1) the patient indicates that they are unable to continue, 2) if the operator determines that the patient is not fit to continue, or 3) the operator assesses that the patient was unable to sustain the speed and cover the distance to the cone prior to the beep sounding [25].

Secondary outcomes

All secondary outcomes will be compared between the PR and usual care arms from baseline to 6-week and 12-week follow-ups.

Health questionnaires will be administered including COPD assessment test (CAT), Clinical COPD questionnaire (CCQ), Hospital Anxiety and Depression Scale (HADS), Patient Health Questionnaire (PHQ-9), Work Productivity and Activity Impairment (WPAI), and European Quality of Life 5-Dimensions (EQ-5D-5L). Pulmonary rehabilitation specific measurements will include the ISWT, Endurance Shuttle Walking Test (ESWT), mid upper arm circumference (MUAC) and sit-to-stand test.

Respiratory symptoms

The CCQ is a simple 10-time validated health related quality of life (HRQoL) questionnaire with good psychometric properties [26]. It consists of 10 items, each scored between 0-6,

For peer review only

divided into three domains (symptoms, functional, mental), with higher scores representing worse HRQoL. The CCQ is responsive to PR with an estimated minimal important improvement of 0.4 [27].

The CAT is a validated, self-administered, short and simple questionnaire that measures HRQoL [28]. The CAT consists of eight items, each scored between 0-5 scored with a range of 0-40; scores of 0-10, 11-20, 21-30, 31-40 representing mild, moderate, severe or very severe negative impact on HRQoL, respectively. The CAT is responsive to the effects of PR with an estimated minimal clinically important difference (MCID) of 2 points [29].

Psychological wellbeing

The HADS questionnaire is a validated, easy to use screening tool for anxiety and depression symptoms in a hospital outpatient setting [30]. The self-report rating scale is composed of 14 items with two 7-item subscales (HADS-Anxiety and HADS-Depression), both ranging from 0-21 with higher scores indicating more severe distress. The HADS is responsive to PR with estimated MCID of 2 points on each subscale [31, 32].

The PHQ-9 is a nine item, validated, short, self-administered, and positively worded questionnaire designed to measure the severity of depression over the last 2 weeks [33]. The total score ranges from 0-27, with high scores indicating high depression, specifically; no depression (0–4), mild (5–9), moderate (10–14), moderately severe (15–19), or severe depression (20–27) [33]. The PHQ-9 has an estimated MCID of 5 points [34].

Work productivity and impairment

The WPAI questionnaire is a validated instrument to measure impairments in work and activities, both paid and unpaid. The WPAI self-administered questionnaire measures time missed from work, impairment of work and regular activities due to overall health and symptoms, during the past seven days [35]. We have added two follow-up supplementary questions, following the WPAI format, to measure productivity with respect to regular household duties in low resource settings.

Health Related Quality of Life

The EQ-5D-5L questionnaire is a standardised questionnaire, developed to measure of health outcomes and defines health in terms of five dimensions: mobility, self-care, usual activities, pain or discomfort and anxiety or depression [36]. The questionnaire also contains a visual analogue scale. The EQ-5D-5L will be used to calculate patient costs per quality adjusted life

For peer review only - http://bmjopen.bmj.com/site/about/guidelines.xhtml

year (QALY). EQ-5D-5L is responsive to change following PR, with a MCID of 0.05 (utility index) and 7.0 (visual analogue scale) [37].

Exercise capacity/ physical function

The five-repetition sit-to-stand test (FTSTS) is a commonly used functional performance measure of lower-limb strength [38]. The FTSTS measures the time taken to stand five times from a sitting position as rapidly as possible. The FTSTS is reliable, valid and responsive to PR with an estimated MCID of 1.7 seconds [39].

The MRC dyspnoea scale is a 5-point self-administered questionnaire based on the sensation of breathing difficulty experienced by the patient during daily life activities. The questionnaire is short, easy to use and has grades ranging from 1 (none) to 5 (almost compete incapacity), with high grades indicating high perceived respiratory disability[40]. The MRC dyspnoea scale is responsive to PR with estimated MCID of 1 points [41, 42].

The ESWT is a constant-load exercise test which measures the ability of the participant to sustain a given sub-maximal exercise capacity; the participant aims to walk at 85% of their maximal ISWT walking speed [23]. The ESWT is frequently used as an exercise tolerance outcome measure for PR. The endpoint of the test is the time the participant walks at the constant endurance speed. The test consists of pre-recorded audio signals at different frequencies giving a total of 16 walking speeds. The ESWT is responsive to PR with MCID following a 6-week PR programme between 174-279 seconds [43].

Physical activity (PA)

Participants will be asked to wear an ActiGraph wGT3X-BT activity monitor (ActiGraph, Pensacola, FL, USA), able to detect a range of PA intensities [44]. Participants will be instructed to wear the PA monitor on the right anterior hip during waking hours for one week prior to attending PR (pre-intervention) and for one week prior to their post-intervention assessment (supplementary Table 2). Written instructions to follow will be provided to the participants prior to wearing and using the PA monitors.

Cost/benefit analysis

The cost of starting and running a PR program will include single and recurrent costs (Table 3). Single payments will include the necessary costs needed to set up and run PR. Recurrent costs refer to any item with a life expectancy of ≤1 year (e.g. disposable materials) [45]. The fixed costs will be captured prior to enrolling the first participant into the PR programme and

For peer review only - http://bmjopen.bmj.com/site/about/guidelines.xhtml

the recurrent costs will be collected at the mid-stage of recruitment. The average fixed and recurrent costs will be calculated separately.

Table 3: Table showing the variables used to calculate fixed and recurrent costs (not an exhaustive list)

Fixed costs	Recurrent costs
• Electrical equipment (laptop, printer, projector)<br>• Equipment for PR (weights, treadmill, cycle ergometer, country-specific equipment, step-up box, chairs)<br>• Equipment for shuttle walking tests (cones, licences, stop watches, tape measure, electrical equipment to play audio)<br>• Equipment for PR assessment (height stadiometer, weight scales, sphygmomanometer, pulse oximeter, spirometer, calibration syringe, country-specific equipment)<br>• Additional safety equipment (blood glucose monitor, Oxygen cylinder holder)<br>• Miscellaneous (filing cabinets, storage units, questionnaire translations, questionnaire licences, staff uniform)<br>• Staff time (creating core PR content including educational material, exercise diaries and other necessary paperwork)	• Staff time to conduct PR (assessment at baseline and discharge, conduct PR classes, telephone calls and data entry)<br>• Disposable equipment (for blood glucose monitor, spirometer mouthpieces, nose-clips)<br>• Servicing costs (spirometer, PR equipment, specifically cycle ergometers)<br>• Miscellaneous (Oxygen cylinders, questionnaire licences, stationery (paper)<br>• Patient costs (transport and meals)

Patient and public involvement (PPI)

Adults with CRDs tell us how they are greatly troubled by breathlessness and express interest in attending a program that can help better manage their condition. They express interest in attending a hospital based programme that allows them to interact with fellow patients. They additionally tell us how the PR programme should be delivered. We have also set up a PPI group at MLI that will meet regularly, and assist with disseminating results following the study.

For peer review only - http://bmjopen.bmj.com/site/about/guidelines.xhtml

Data analysis

All randomized participants will be included in the intention to treat analysis population. The primary efficacy analysis will be based on both per protocol and modified intention-to-treat populations. For the primary analysis, the differences in the primary outcome (walking distance on the ISWT) with the corresponding two-sided 95% confidence interval and p-value will be estimated using a stratified analysis; a p-value <0.05 will be the measure for statistical significance. Predictive analytics software (SPSS; Statistical Package for the Social Sciences) will be used to analyse the data. Continuous data will be presented as mean and standard deviation or median and interquartile ranges, whilst categorical data will be presented as frequencies and percentages. All data will be assessed for normality and appropriate parametric and non-parametric tests will be used. Categorical variables between the two treatment groups will be compared using chi-square and Fisher exact test as appropriate. Continuous variables will be compared using t-test for normally distributed data and Mann-Whitney-U test for non-normally distributed data. Any baseline differences will be adjusted for. Both intention-to-treat and per-protocol analyses will be conducted after imputing any missing data. There will be no formal interim analysis of data. The final analysis will be performed when all the 114 participants have completed the last study related visit or previously withdrawn from the trial. We will fit linear mixed models for both per protocol and intention to treat analyses.

Data management

An Independent Data Monitoring Committee will be established at the University of Leicester, UK to review high level safety data (serious adverse events and adverse events) at least quarterly, and as needed on an ad hoc basis to ensure the continuing safety of the participants enrolled in this study.

All data collected during the trial will be entered into the Research Electronic Data Capture (REDCap) [46, 47] with access via a secure password protected web-interface hosted by the University of Leicester, UK. Study participants will be assigned a study-specific identification code.

Ethics and dissemination

The study received ethical approvals from the University of Leicester research ethics committee (United Kingdom) (Ref No. 22349) and locally from the Mulago Hospital Research and Ethics Committee (MHREC1478), Kampala, Uganda as well as the Uganda National Council for Science and Technology (SS5105).

For peer review only - http://bmjopen.bmj.com/site/about/guidelines.xhtml

Confidentiality

The confidentiality of all participants will be protected to the fullest extent possible. All patient information will be kept secure and will be available only to the treatment staff and representatives of the sponsors, regulators, and ethics committees.

All participants will be provided with a unique identification number which will be recorded in the participant enrolment log and stored in a secure place. Study participants will not be identified by name on any case report form, email or on any other documentation sent to the central database and will not be reported by name in any report, presentation or publication resulting from data collected in this study. Participants' data/specimens will be identified by study number or hospital number only.

Dissemination

Results of the study will be published in peer-reviewed journals and findings disseminated at appropriate local, regional and international scientific meetings and conferences. Social media will be used to disseminate information and summaries of results to a wider public domain. Furthermore, a participant dissemination meeting will be held following this trial, in which study participants will receive a summary of the findings.

COVID-19 provisions

Modifications will be made to the delivery of the PR program due to the Corona Virus Disease 2019 (COVID-19) pandemic. The PR room will be re-organized to allow for social distancing (minimum 2-meters) for both study staff and study participants. The maximum number of participants participating in the PR session will be reduced from 12 to 8 to ensure social distancing between participants. Before accessing the PR room, all participants and staff will be required to undergo temperature measurement using a hand-held non-contact thermometer, wash hands with soap or alcohol-based hand sanitizer. All participants will be provided with face masks during PR sessions. All surfaces inside the PR room will be disinfected before and after every PR session. PR sessions will be conducted in the morning hours to allow participants travel back home in time before the evening rush hour and the standard operating procedure for data collection will be modified ensure 2-meter distancing between the study staff and study participant. Study participants will undergo COVID-19 testing before starting PR and as needed during the hospital based sessions. All study staff will be required to wear

For peer review only - http://bmjopen.bmj.com/site/about/guidelines.xhtml

N95 masks at all times and will undergo COVID-19 training with emphasis on infection prevention and control, and screening study participants for signs and symptoms of the disease.

Figure legend

Figure 1: Figure showing the study flow in the Post TB Pulmonary Rehabilitation trial

Legend: *PTBLD-post-TB lung disease; PR – Pulmonary Rehabilitation; R&A – Recruitment and Assessment*

Contributors

SJS is the principal investigator of the Global RECHARGE project while BK is the in-country principal investigator. WK, MWO, AVJ, RK, RM, AM and RCF have been involved in drafting the work and revising it critically for important intellectual content. AB, RJ, MCS and JM have substantially contributed to the development of the intervention and the design of the trial. All authors have revised the content and approved the final version to be published.

Competing interests

None declared

Funding and acknowledgments

This research was funded by the National Institute for Health Research (NIHR) (17/63/20) using UK aid from the UK Government to support global health research. The views expressed in this publication are those of the author(s) and not necessarily those of the NIHR or the UK Department of Health and Social Care.

Data sharing

Following the completion of the project, data from the Global Health Research Group on Respiratory Rehabilitation (Global RECHARGE) Core Dataset [16] will be made available to the wider community upon reasonable request.

For peer review only - http://bmjopen.bmj.com/site/about/guidelines.xhtml

References

1. WHO, *Global tuberculosis report 2019.* 2019.
2. Dye C, H.A., Maher D, S. Hosseini SM, Nkhoma W, and Salaniponi FM., *Disease and Mortality in Sub-Saharan Africa. 2nd edition. Chapter 13. Tuberculosis*. 2006.
3. Pasipanodya, J.G., et al., *Pulmonary impairment after tuberculosis.* Chest, 2007. **131**(6): p. 1817-1824.
4. Harries, A.D., et al., *Successfully treated but not fit for purpose: paying attention to chronic lung impairment after TB treatment.* The International Journal of Tuberculosis and Lung Disease, 2016. **20**(8): p. 1010-1014.
5. van Kampen, S.C., et al., *International research and guidelines on post-tuberculosis chronic lung disorders: a systematic scoping review.* BMJ Global Health, 2018. **3**(4): p. e000745.
6. Chakaya, J., B. Kirenga, and H. Getahun, *Long term complications after completion of pulmonary tuberculosis treatment: A quest for a public health approach.* Journal of Clinical Tuberculosis and Other Mycobacterial Diseases, 2016. **3**: p. 10-12.
7. Nici, L., et al., *American thoracic society/European respiratory society statement on pulmonary rehabilitation.* American journal of respiratory and critical care medicine, 2006. **173**(12): p. 1390-1413.
8. Allwood, B., et al., *Post-tuberculosis lung health: perspectives from the First International Symposium.* 2020. **24**(8): p. 820-828.
9. Visca, D., et al., *Post-tuberculosis sequelae: the need to look beyond treatment outcome.* 2020. **24**(8): p. 761-762.
10. Lacasse, Y., et al., *Meta-analysis of respiratory rehabilitation in chronic obstructive pulmonary disease. A Cochrane systematic review*. 2007.
11. McCarthy, B., et al., *Pulmonary rehabilitation for chronic obstructive pulmonary disease.* Cochrane database of systematic reviews, 2015(2).
12. Bolton, C.E., et al., *British Thoracic Society guideline on pulmonary rehabilitation in adults: accredited by NICE.* Thorax, 2013. **68**(Suppl 2): p. ii1-ii30.
13. Spruit, M.A., et al., *An official American Thoracic Society/European Respiratory Society statement: key concepts and advances in pulmonary rehabilitation.* American journal of respiratory and critical care medicine, 2013. **188**(8): p. e13-e64.
14. Jones, R., et al., *A pre–post intervention study of pulmonary rehabilitation for adults with post-tuberculosis lung disease in Uganda.* International Journal of Chronic Obstructive Pulmonary Disease, 2017. **12**: p. 3533.
15. Jones, R., et al., *A development study of pulmonary rehabilitation for patients with chronic lung disease in Uganda*. 2016, Eur Respiratory Soc.
16. Orme, M.W., M. Orme, and R.J.J.o.G.H. Free, *Global RECHARGE: Establishing a standard international data set for pulmonary rehabilitation in low-and middle-income countries.* 2020. **10**(2).
17. Graham, B.L., et al., *Standardization of spirometry 2019 update. An official American thoracic society and European respiratory society technical statement.* 2019. **200**(8): p. e70-e88.
18. Evans, R.A. and S.J. Singh, *Minimum important difference of the incremental shuttle walk test distance in patients with COPD.* Thorax, 2019. **74**(10): p. 994.
19. GOLD, *Global strategy for the diagnosis, management, and prevention of chronic obstructive pulmonary disease, 2020 report.* 2020.
20. Powers, S.K., S.L. Dodd, and E.M. Jackson, *Total fitness & wellness*. 2013: Pearson Higher Ed.
21. Borg, G., *Perceived exertion as an indicator of somatic stress.* Scandinavian journal of rehabilitation medicine, 1970.
22. Borg, G.A., *Psychophysical bases of perceived exertion.* Medicine & science in sports & exercise, 1982.

For peer review only - http://bmjopen.bmj.com/site/about/guidelines.xhtml

23. Revill, S., et al., *The endurance shuttle walk: a new field test for the assessment of endurance capacity in chronic obstructive pulmonary disease.* Thorax, 1999. **54**(3): p. 213-222.

24. Singh, S.J., et al., *Minimum clinically important improvement for the incremental shuttle walking test.* Thorax, 2008. **63**(9): p. 775.

25. Singh, S.J., et al., *Development of a shuttle walking test of disability in patients with chronic airways obstruction.* Thorax, 1992. **47**(12): p. 1019-1024.

26. Van der Molen, T., et al., *Development, validity and responsiveness of the Clinical COPD Questionnaire.* Health and quality of life outcomes, 2003. **1**(1): p. 13.

27. Kon, S.S., et al., *The Clinical COPD Questionnaire: response to pulmonary rehabilitation and minimal clinically important difference.* Thorax, 2014. **69**(9): p. 793-798.

28. Jones, P., et al., *Development and first validation of the COPD Assessment Test.* European Respiratory Journal, 2009. **34**(3): p. 648-654.

29. Kon, S.S., et al., *Minimum clinically important difference for the COPD Assessment Test: a prospective analysis.* The Lancet Respiratory Medicine, 2014. **2**(3): p. 195-203.

30. Nowak, C., et al., *Accuracy of the hospital anxiety and depression scale for identifying depression in chronic obstructive pulmonary disease patients.* Pulmonary medicine, 2014. **2014**.

31. Smid, D.E., et al., *Responsiveness and MCID estimates for CAT, CCQ, and HADS in patients with COPD undergoing pulmonary rehabilitation: a prospective analysis.* Journal of the American Medical Directors Association, 2017. **18**(1): p. 53-58.

32. Wynne, S., et al., *The Hospital Anxiety and Depression Scale (HADS) in Bronchiectasis: Response to pulmonary rehabilitation (PR) and Minimum Clinically Important Difference (MCID).* 2019, Eur Respiratory Soc.

33. Kroenke, K. and R.L. Spitzer, *The PHQ-9: a new depression diagnostic and severity measure.* Psychiatric annals, 2002. **32**(9): p. 509-515.

34. Löwe, B., et al., *Monitoring depression treatment outcomes with the patient health questionnaire-9.* Medical care, 2004: p. 1194-1201.

35. Reilly, M.C., A.S. Zbrozek, and E.M. Dukes, *The validity and reproducibility of a work productivity and activity impairment instrument.* Pharmacoeconomics, 1993. **4**(5): p. 353-365.

36. Group, T.E., *EuroQol-a new facility for the measurement of health-related quality of life.* Health policy, 1990. **16**(3): p. 199-208.

37. Nolan, C.M., et al., *The EQ-5D-5L health status questionnaire in COPD: validity, responsiveness and minimum important difference.* Thorax, 2016. **71**(6): p. 493-500.

38. Bohannon, R.W., *Sit-to-stand test for measuring performance of lower extremity muscles.* Perceptual and motor skills, 1995. **80**(1): p. 163-166.

39. Jones, S.E., et al., *The five-repetition sit-to-stand test as a functional outcome measure in COPD.* Thorax, 2013. **68**(11): p. 1015-1020.

40. Stenton, C., *The MRC breathlessness scale.* Occupational Medicine, 2008. **58**(3): p. 226-227.

41. De Torres, J.P., et al., *Power of outcome measurements to detect clinically significant changes in pulmonary rehabilitation of patients with COPD.* Chest, 2002. **121**(4): p. 1092-1098.

42. Crisafulli, E. and E.M. Clini, *Measures of dyspnea in pulmonary rehabilitation.* Multidisciplinary respiratory medicine, 2010. **5**(3): p. 202.

43. Zatloukal, J., et al., *The minimal important difference for the endurance shuttle walk test in individuals with chronic obstructive pulmonary disease following a course of pulmonary rehabilitation.* Chronic Respiratory Disease, 2019. **16**: p. 1479973119853828.

44. Bassett, D.R. and D. John, *Use of pedometers and accelerometers in clinical populations: validity and reliability issues.* Physical therapy reviews, 2010. **15**(3): p. 135-142.

45. Lucas, A.O. and H.M. Gilles, *Short textbook of public health medicine for the tropics. Chapter-Approaches to economic evaluation*. 2003: CRC Press.

For peer review only - http://bmjopen.bmj.com/site/about/guidelines.xhtml

46. Harris, P.A., et al., *Research electronic data capture (REDCap)—a metadata-driven methodology and workflow process for providing translational research informatics support.* Journal of biomedical informatics, 2009. **42**(2): p. 377-381.
47. Harris, P.A., et al., *The REDCap consortium: Building an international community of software platform partners.* Journal of biomedical informatics, 2019. **95**: p. 103208.

For peer review only

For peer review only - http://bmjopen.bmj.com/site/about/guidelines.xhtml

Table 1: The table shows the assessment and follow up schedule

Observation/Investigation	Screening/ Baseline assessments	Randomisation	12 weeks of study participation	
			Hospital based Pulmonary Rehab	Follow-up phase of homebased exercises
			End of 6 weeks of PR	End of 6 weeks of home exercises
Written informed consent	x	x		
Demographics	x	x		
Medical history	x	x		
Clinical exam	x	x		
Chest X-ray	x			
Spirometry		x		
MRC dyspnoea grade	x		x	x
Assess symptoms	x		x	x
Incremental Shuttle Walk Test	x		x	x
Endurance Shuttle Walk Test	x		x	x
Borg breathlessness scale	x		x	x
Mid Upper Arm Circumference	x		x	x
Sit-to-stand time	x		x	x
COPD Assessment Test		x	x	x
Clinical COPD Questionnaire		x	x	x
Patient Health Questionnaire		x	x	x
HADS		x	x	x
WPAI		x	x	x
Physical Activity (Actigraph monitor)		x	x	x
Cost/Benefit Analysis		x	x	x
EQ-5D-5L Questionnaire		x	x	x

For peer review only

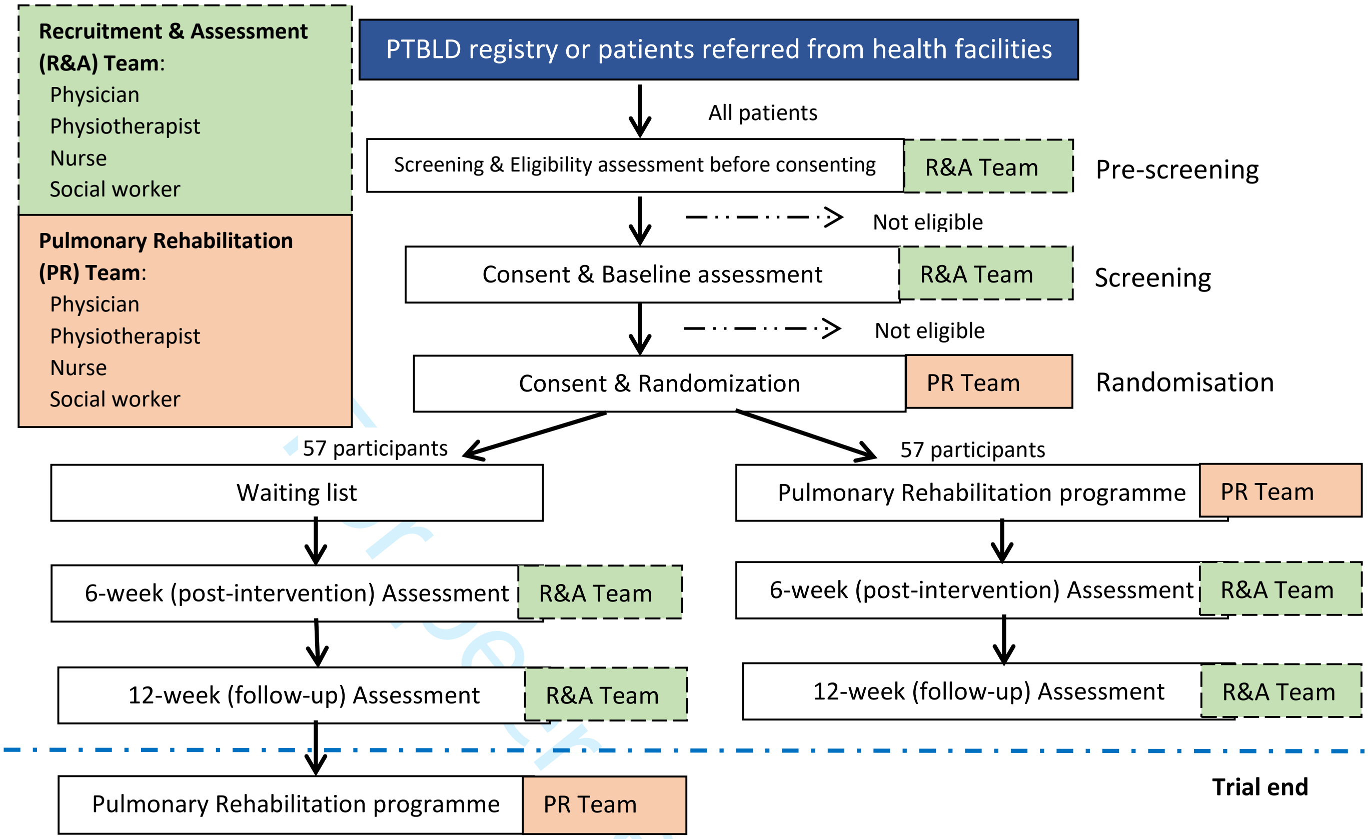

Figure 1: Figure showing the study flow in the Post TB Pulmonary Rehabilitation trial

Legend: *PTBLD-post-TB lung disease; PR – Pulmonary Rehabilitation; R&A – Recruitment and Assessment*

Supplementary Table 1: A list of warm up and cool down activities during PR session

1. Marching on the spot, slowly bringing the feet off the floor for up to 1 minute
2. Heel digs: alternate heel digs in front of the body with toes pointing to the ceiling, add in a biceps curl (repeat 10-12 times).
3. Toe taps: Tap the toes to the floor in front of alternating legs at a comfortable distance. Heels stay off the ground (repeat 10-12 times).
4. High knee marching – with opposite hand to opposite knee.
5. Side bends: With arms relaxed by your side, leaning over to the right for 8 to 10 seconds and back to centre, then lean to the left for 8 to 10 seconds and back to the centre (repeat 8 times).
6. Arms stretched up, forwards and down.
7. Alternate punching of arms forward.
8. Seated or standing side taps to the floor with the foot – alternate legs.
9. Seated or standing in upright posture, feet placed shoulder breadth apart – shoulder roll in both directions (clockwise and anti-clockwise).
10. Seated or standing in upright posture, feet placed shoulder breath apart, elbows bent with hands onto shoulder – elbows make circles in clockwise and anti-clockwise.
11. Hamstring stretch: With right leg straight, place it in front of the body, heel pushed into the floor with toes pointing toward the ceiling. Slightly bend the left knee, place hands on the straight right leg and gently lean forward. Hold the stretch for 10-15 seconds then return to upright position. Repeat on left leg.
12. Quadriceps stretch: While holding a chair or onto a wall, stand on your left leg and grab your right foot using your right hand, pulling it gently towards the ceiling. Hold the position for 10-15 seconds and return to upright position and repeat on the right leg.

PR – Pulmonary Rehabilitation

Supplementary Table 2: Physical activity data collection and accelerometry processing criteria

Criteria	Details
**Accelerometer Model**	ActiGraph wGT3X-BT (version 6.13.4; firmware 1.9.2)
**Serial number range**	Twenty unique devices will be used; ranging from MOS2E09190617 to MOS2E25190750 and averaging six deployments per device (same serial used for baseline and follow-up wear periods to remove any inter-device variability)
**Piezosensor orientation**	Triaxial
**Mode setup**	Mode 29 (x, y, z, steps, lux)
**Original sample rate**	100 Hz (.gt3x file format)
**Deployment method**	Baseline:<br>Fitted by research team on Day 0 (Baseline PR Assessment)<br>Fitted by participant on Day 1<br>Follow-up:<br>Fitted by research team Day 0 (11th/12th session)<br>Fitted by participant on Day 1
**Location worn**	Anterior hip adjacent to the mid-line of the thigh
**Requested days of wear**	7 days of free-living (10,080 epochs)
**Initialization**	Not deployed in delay mode in order to standardised capture of Day 0 (00:00) with stop time based on date of first PR class (baseline) and date of follow-up assessment
**Wear instructions**	Wear continuously except for sleep and water-based activities
**Non-wear appropriation**	≥60 min of consecutive 0s with allowance for 2 minutes of interruptions
**Valid day criteria**	≥8 hours of valid waking wear time
**Valid file**	≥4 valid days for each of the two time points
**Missing data**	Data modelling or imputation will not be performed
**Epoch length**	60 seconds
**Intensity classification (absolute)**	Uniaxial (x-axis) intensity cut-points as follows: Stationary <100 cpm; Light 100-2019 cpm; Moderate 2020-5998cpm; Vigorous ≥5999cpm (Moderate-to-vigorous ≥2020 cpm)
**Intensity classification (relative)**	Uniaxial (x-axis) cut-points based on Endurance Shuttle Walk Test performance

PR – Pulmonary Rehabilitation

SPIRIT

STANDARD PROTOCOL ITEMS: RECOMMENDATIONS FOR INTERVENTIONAL TRIALS

SPIRIT 2013 Checklist: Recommended items to address in a clinical trial protocol and related documents*

Section/item	Item No	Description	Addressed on page number
**Administrative information**			
Title	1	Descriptive title identifying the study design, population, interventions, and, if applicable, trial acronym	1
Trial registration	2a	Trial identifier and registry name. If not yet registered, name of intended registry	3
	2b	All items from the World Health Organization Trial Registration Data Set	N/A
Protocol version	3	Date and version identifier	1
Funding	4	Sources and types of financial, material, and other support	1,16
Roles and responsibilities	5a	Names, affiliations, and roles of protocol contributors	1
	5b	Name and contact information for the trial sponsor	1,16
	5c	Role of study sponsor and funders, if any, in study design; collection, management, analysis, and interpretation of data; writing of the report; and the decision to submit the report for publication, including whether they will have ultimate authority over any of these activities	N/A
	5d	Composition, roles, and responsibilities of the coordinating centre, steering committee, endpoint adjudication committee, data management team, and other individuals or groups overseeing the trial, if applicable (see Item 21a for data monitoring committee)	14
**Introduction**			

For peer review only

For peer review only - http://bmjopen.bmj.com/site/about/guidelines.xhtml

Background and rationale	6a	Description of research question and justification for undertaking the trial, including summary of relevant studies (published and unpublished) examining benefits and harms for each intervention	4
	6b	Explanation for choice of comparators	6,7,8
Objectives	7	Specific objectives or hypotheses	4
Trial design	8	Description of trial design including type of trial (eg, parallel group, crossover, factorial, single group), allocation ratio, and framework (eg, superiority, equivalence, noninferiority, exploratory)	4

Methods: Participants, interventions, and outcomes

Study setting	9	Description of study settings (eg, community clinic, academic hospital) and list of countries where data will be collected. Reference to where list of study sites can be obtained	5
Eligibility criteria	10	Inclusion and exclusion criteria for participants. If applicable, eligibility criteria for study centres and individuals who will perform the interventions (eg, surgeons, psychotherapists)	5,6
Interventions	11a	Interventions for each group with sufficient detail to allow replication, including how and when they will be administered	8,9
	11b	Criteria for discontinuing or modifying allocated interventions for a given trial participant (eg, drug dose change in response to harms, participant request, or improving/worsening disease)	N/A
	11c	Strategies to improve adherence to intervention protocols, and any procedures for monitoring adherence (eg, drug tablet return, laboratory tests)	8,9,10
	11d	Relevant concomitant care and interventions that are permitted or prohibited during the trial	N/A

For peer review only - http://bmjopen.bmj.com/site/about/guidelines.xhtml

Outcomes	12	Primary, secondary, and other outcomes, including the specific measurement variable (eg, systolic blood pressure), analysis metric (eg, change from baseline, final value, time to event), method of aggregation (eg, median, proportion), and time point for each outcome. Explanation of the clinical relevance of chosen efficacy and harm outcomes is strongly recommended	10,11,12
Participant timeline	13	Time schedule of enrolment, interventions (including any run-ins and washouts), assessments, and visits for participants. A schematic diagram is highly recommended (see Figure)	7,20
Sample size	14	Estimated number of participants needed to achieve study objectives and how it was determined, including clinical and statistical assumptions supporting any sample size calculations	7
Recruitment	15	Strategies for achieving adequate participant enrolment to reach target sample size	5
**Methods: Assignment of interventions (for controlled trials)**			
Allocation:			
Sequence generation	16a	Method of generating the allocation sequence (eg, computer-generated random numbers), and list of any factors for stratification. To reduce predictability of a random sequence, details of any planned restriction (eg, blocking) should be provided in a separate document that is unavailable to those who enrol participants or assign interventions	6
Allocation concealment mechanism	16b	Mechanism of implementing the allocation sequence (eg, central telephone; sequentially numbered, opaque, sealed envelopes), describing any steps to conceal the sequence until interventions are assigned	6
Implementation	16c	Who will generate the allocation sequence, who will enrol participants, and who will assign participants to interventions	6
Blinding (masking)	17a	Who will be blinded after assignment to interventions (eg, trial participants, care providers, outcome assessors, data analysts), and how	7

	17b	If blinded, circumstances under which unblinding is permissible, and procedure for revealing a participant's allocated intervention during the trial	N/A
**Methods: Data collection, management, and analysis**			
Data collection methods	18a	Plans for assessment and collection of outcome, baseline, and other trial data, including any related processes to promote data quality (eg, duplicate measurements, training of assessors) and a description of study instruments (eg, questionnaires, laboratory tests) along with their reliability and validity, if known. Reference to where data collection forms can be found, if not in the protocol	14
	18b	Plans to promote participant retention and complete follow-up, including list of any outcome data to be collected for participants who discontinue or deviate from intervention protocols	14
Data management	19	Plans for data entry, coding, security, and storage, including any related processes to promote data quality (eg, double data entry; range checks for data values). Reference to where details of data management procedures can be found, if not in the protocol	14
Statistical methods	20a	Statistical methods for analysing primary and secondary outcomes. Reference to where other details of the statistical analysis plan can be found, if not in the protocol	13,14
	20b	Methods for any additional analyses (eg, subgroup and adjusted analyses)	N/A
	20c	Definition of analysis population relating to protocol non-adherence (eg, as randomised analysis), and any statistical methods to handle missing data (eg, multiple imputation)	13,14
**Methods: Monitoring**			
Data monitoring	21a	Composition of data monitoring committee (DMC); summary of its role and reporting structure; statement of whether it is independent from the sponsor and competing interests; and reference to where further details about its charter can be found, if not in the protocol. Alternatively, an explanation of why a DMC is not needed	14
	21b	Description of any interim analyses and stopping guidelines, including who will have access to these interim results and make the final decision to terminate the trial	14

Harms	22	Plans for collecting, assessing, reporting, and managing solicited and spontaneously reported adverse events and other unintended effects of trial interventions or trial conduct	14
Auditing	23	Frequency and procedures for auditing trial conduct, if any, and whether the process will be independent from investigators and the sponsor	N/A
**Ethics and dissemination**			
Research ethics approval	24	Plans for seeking research ethics committee/institutional review board (REC/IRB) approval	14
Protocol amendments	25	Plans for communicating important protocol modifications (eg, changes to eligibility criteria, outcomes, analyses) to relevant parties (eg, investigators, REC/IRBs, trial participants, trial registries, journals, regulators)	N/A
Consent or assent	26a	Who will obtain informed consent or assent from potential trial participants or authorised surrogates, and how (see Item 32)	5
	26b	Additional consent provisions for collection and use of participant data and biological specimens in ancillary studies, if applicable	N/A
Confidentiality	27	How personal information about potential and enrolled participants will be collected, shared, and maintained in order to protect confidentiality before, during, and after the trial	14,15
Declaration of interests	28	Financial and other competing interests for principal investigators for the overall trial and each study site	16
Access to data	29	Statement of who will have access to the final trial dataset, and disclosure of contractual agreements that limit such access for investigators	16
Ancillary and post-trial care	30	Provisions, if any, for ancillary and post-trial care, and for compensation to those who suffer harm from trial participation	N/A

For peer review only

Dissemination policy	31a	Plans for investigators and sponsor to communicate trial results to participants, healthcare professionals, the public, and other relevant groups (eg, via publication, reporting in results databases, or other data sharing arrangements), including any publication restrictions	15
	31b	Authorship eligibility guidelines and any intended use of professional writers	16
	31c	Plans, if any, for granting public access to the full protocol, participant-level dataset, and statistical code	16
**Appendices**			
Informed consent materials	32	Model consent form and other related documentation given to participants and authorised surrogates	Attached
Biological specimens	33	Plans for collection, laboratory evaluation, and storage of biological specimens for genetic or molecular analysis in the current trial and for future use in ancillary studies, if applicable	N/A

*It is strongly recommended that this checklist be read in conjunction with the SPIRIT 2013 Explanation & Elaboration for important clarification on the items. Amendments to the protocol should be tracked and dated. The SPIRIT checklist is copyrighted by the SPIRIT Group under the Creative Commons "Attribution-NonCommercial-NoDerivs 3.0 Unported" license.

For peer review only
